# GLUT1 and Cerebral Glucose Hypometabolism in Human Focal Cortical Dysplasia Is Associated with Hypermethylation of Key Glucose Regulatory Genes

**DOI:** 10.1007/s12035-025-04871-z

**Published:** 2025-04-07

**Authors:** Chaitali Ghosh, Rosemary Westcott, David Skvasik, Ishant Khurana, Jean Khoury, Ingmar Blumcke, Assam El-Osta, Imad M. Najm

**Affiliations:** 1 Neurovascular Research, Department of Biomedical Engineering, Lerner Research Institute, Cleveland Clinic, 9500 Euclid Avenue, Cleveland, OH 44195, USA; 2 Department of Biomedical Engineering and Molecular Medicine, Cleveland Clinic Lerner College of Medicine of Case Western Reserve University, Cleveland, OH, USA; 3 Epigenetics in Human Health and Disease Program, Baker Heart and Diabetes Institute, Melbourne, Australia; 4 Epilepsy Center, Neurological Institute, Cleveland Clinic, Cleveland, OH, USA; 5 Institute of Neuropathology, University Hospitals Erlangen, Erlangen, Germany

**Keywords:** Focal cortical dysplasia, Drug-resistant epilepsy, Biomarker, Glucose transporter-1, DNA methylation, Neurotrophic factors, Angiogenic marker

## Abstract

Focal cortical dysplasia (FCD) is a significant etiological factor in drug-resistant epilepsy, linked with disturbances in neurovascular metabolism. Our study investigated regulation of glucose-transporter1 (GLUT1) and cerebral hypometabolism within FCD subtypes. Surgically excised human brain specimens underwent histopathological categorization. A subset of samples was assessed for DNA methylation changes of glucose metabolism-related genes. We evaluated GLUT1, vascular endothelial growth factor alpha (VEGFα), monocarboxylate-transporter (MCT2), and mammalian target of rapamycin (mTOR) expression, measured glucose-lactate concentrations, and established correlations with patients’ demographic and clinical profiles. Furthermore, we investigated the impact of DNA methylation inhibitor decitabine and hypometabolic condition on the uptake of [^3^H]-2-deoxyglucose and ATPase in epileptic-brain endothelial cells (EPI-EC). We observed hypermethylation of GLUT1 and glucose metabolic genes in FCD brain/blood samples and could distinguish FCDIIa/b from mild malformations of cortical development (mMCD), with oligodendroglial hyperplasia (MOGHE) and non-lesional brains. Low GLUT1 and glucose-lactate ratios corresponded to elevated VEGFα and MCT2 in FCDIIa/b vs. non-lesional tissues, independent of age, gender, seizure-onset, or duration of epilepsy. Increased mTOR-signaling in FCDIIa/b tissues was evident. Decitabine stimulation increased GLUT1, decreased VEGFα expression, restored glucose uptake and ATPase activity in EPI-ECs, and reduced mTOR and MCT2 levels in human embryonic-kidney cells. We demonstrated: hypermethylation of glucose regulatory genes distinguish FCDIIa/b from mMCD, MOGHE and non-lesional types, glucose uptake reduction is due to GLUT1 suppression mediated possibly by a GLUT1-mTOR mechanism; and DNA methylation regulates cellular glucose uptake and metabolism. Together, these studies may lead to GLUT1-mediated biomarkers and identify early intervention strategies in FCD.

## Introduction

Epilepsy is a complex neurological disease, and focal cortical dysplasia (FCD) is a common cause of pharmacoresistant epilepsy [1–4]. Fluoro-deoxy-D-glucose PET radiotracer is routinely used for imaging epileptic foci by localizing areas of focal cerebral hypometabolism [5]. Glucose normally enters the brain by facilitated transport via a glucose transporter (GLUT1/*SLC2A1*) located on the luminal and abluminal surfaces of the blood–brain barrier (BBB) [6, 7]. GLUT1 is responsible for mediating more than 90% of glucose transport across the brain. Notably, its presence is often markedly reduced or absent in FCD regions [5, 8–10].

In cortical dysplasia (CD) rodent model brains, we found decreased GLUT1, elevated mammalian target of rapamycin (mTOR) and monocarboxylate transporter 2 (MCT2) expression, and persistent imbalance in glucose-lactate levels upon seizure induction [9]. We further identified increased vessel density which could be due to angiogenesis and BBB dysfunction with decreased cortical tight junction proteins, sustained longer in CD rats post seizure induction. However, the epigenetic regulation by DNA methylation and characterization of GLUT1 pattern in FCD subtypes and the key regulatory proteins responsible for glucose metabolism is presently understudied and unknown.

Decitabine (5-Aza-2′-deoxycytidine, 5Aza) is a nucleoside analog that incorporates into replicating DNA in place of cytosine, traps and promotes the degradation of DNA methyltransferases, therefore acting as a DNA methylation inhibitor [11, 12]. 5Aza has proven beneficial for cancer therapy as an FDA-approved drug [13–15]. Hence, by assessing 5Aza as a *proof-of-concept*, the DNA demethylation approach may elucidate the functional impact of methylation on the functionality of GLUT1 and the broader glucose metabolic pathway.

This study investigates the regulatory dynamics of GLUT1 and cerebral hypometabolism across various FCD subtypes (such as FCDIIa, FCDIIb, mMCD, and MOGHE) and non-lesional resected tissue, which may pave the way for early diagnostic and therapeutic strategies. We show surgically excised brain tissue (matched with blood samples) from patients with pharmacoresistant epilepsy to enhance the identification of DNA methylation biomarkers for glucose regulation. Additionally, the study assessed the principal glucose regulatory proteins and the glucose/lactate ratios in FCDIIa/b versus non-lesional brain tissues. Correlations were drawn between the metabolic levels of glucose in the brain, protein expressions, and the demographic/clinical attributes of the participants, including age, gender, age at seizure onset, and epilepsy duration. The influence of DNA hypermethylation on these factors is further substantiated through the application of the DNA methylation inhibitor decitabine in EPI-ECs and HEK cells, evaluating GLUT1, VEGFα levels, the uptake pattern of [^3^H]-2-deoxyglucose, ATPase activity, and mTOR signaling pathways.

## Materials and Methods

### Human Subjects

Resected human brain tissues from a deidentified homogenous population of male and female patients with medically refractory epilepsy (*n* = 57) which were histopathologically well characterized: (1) using the ILAE classification [16] into FCD subtypes: FCDIIa, FCDIIb, mMCD, MOGHE, and non-lesional neocortical areas, *n* = 54 (Table S1). We utilized a subset of matched brain and blood samples (*n* = 15) under IRB 12–1000. (2) A cohort of paired epileptic and non-epileptic tissues from same brain (*n* = 3, Table S1) pre-characterized through non-invasive evaluation (scalp-video-EEG monitoring, magnetic resonance imaging, and positron emission tomography), invasive recordings (stereoelectroencephalography), and post-resective histopathological examination under IRB 07–322. We obtained written informed consent from each patient under Cleveland Clinic Institutional Review Board-approved protocols (IRB 07–322 and IRB 12–1000). In this retrospective study, we randomly selected (1) pediatric and adult patients with pharmacoresistant epilepsy who underwent resective surgery following minimally invasive or non-invasive evaluation, and (2) surgically excised human brain specimens that underwent professional histopathological categorization into distinct FCD subtypes and non-lesional. Among non-lesional epilepsy, eleven patients underwent invasive monitoring to more precisely delineate the epileptogenic zone. These cases were presented at the Cleveland Clinic Epilepsy Center’s Patient Management Conference, where experts from the center discuss complex cases of medically intractable epilepsy in surgical candidates. The epileptogenic zone was identified based on a thorough review of non-invasive testing, including imaging, scalp EEG, functional imaging, and invasive EEG data. The decision to recommend resection of specific brain regions was made following this collaborative discussion. Although the resected tissue was from areas likely corresponding to the epileptogenic zone, histopathological analysis did not show evidence of FCD. This finding aligns with the newly introduced subgroup in the recent international FCD classification [16]. Outcome data for all patients are provided in Table S1. The patients with dual pathology were excluded. These study criteria were explicitly chosen to focus on epilepsy patients with varied FCD subtypes and diagnosed with refractory seizures and which could be later expanded to a broader FCD patient population.

### DNA Methylation

*Methylation sequencing (Methyl-seq)*- DNA extractions were obtained from a subset of matched brain tissues and blood samples of FCD subtypes (*n* = 15) and compared using methyl-capture [17, 18]. Genomic DNA was fragmented by sonication, and 500 ng of genomic DNA was used for methyl-CpG enrichment using MethylMiner (Life Technologies), with a positive control methylated DNA spike-in option. Eluted DNA was then quantified, and 10 ng of this methylated DNA was used to generate Illumina sequencing libraries via DNA Library Preparation Kit [17, 18]. Regions of DNA methylation changes were annotated on the UCSC genome browser and expressed as observed differences in DNA methylation for each group. After data quality control, sequenced reads were aligned to the human reference genome and then counted. Profiles of DNA methylation were compared between all pairwise combinations of samples using the MACS peak-calling software. The number of reads aligning to each region (peak) was counted and annotated producing a count matrix (reads/region/sample). Differentially methylated regions of GLUT1/*SLC2A1*, MCT2/*SLC16A7*, *VEGFα*, and *MTOR* genes were identified by using edgeR with a generalized linear model.

### Tissue Lysate Preparation and Western Blot

Approximately 50 mg of fresh-frozen human brain cortical tissue (*n* = 49) was lysed with radioimmunoprecipitation assay buffer (Sigma-Aldrich, #R0278) [19, 20]. Protein concentration was estimated by the Bradford method. Tissue lysates were separated by 8% or 12% sodium dodecyl sulfate polyacrylamide gel electrophoresis (SDS-PAGE) and transferred to polyvinylidene fluoride membranes by semi-dry transfer, then blocked and probed with the primary antibody followed by the appropriate secondary antibody as previously described [9, 20]. Protein expression was normalized by β-actin as loading controls, and the densitometry quantification of the images was performed using ImageJ software (see Methods S1 for details).

### Glucose-Lactate Measurement

Glucose and lactate were measured in human brain tissue samples via a dual-channel immobilized oxidase enzyme analyzer as previously described [9]. The sample supernatants were analyzed for glucose and lactate levels measured in mmol/liter, and the data plotted as glucose/lactate ratio (details in Methods S1).

### Primary Brain Endothelial Cell and Human Embryonic Kidney Cell Culture

We used primary microvascular endothelial cells (EPI-EC, *n* = 3) derived from brain specimens resected from patients with pharmacoresistant epilepsy as described earlier [19] and Methods S1. Primary control Human Brain Microvascular Endothelial cells (HBMECs) were purchased from Cell Systems (#ACBRI 376). HBMECs were dissociated from normal human brain cortical tissue obtained from healthy donors and characterized by von-Willebrand factor staining. Human Embryonic Kidney (HEK)293 cells were obtained from ATCC (#CRL-1573) [9].

### Cell Treatment with Low Glucose-High Lactate and DNA Methylation Inhibitor, Decitabine/5-Aza-2′-Deoxycytidine (5Aza) and Western Blot

Both cell types (EPI-EC and HEK) in the treatment group were cultured separately in 100-mm Petri dishes in complete medium containing 1 mM glucose with 20 mM lactate (Sigma-Aldrich, USA, #71718) for 24 h to mimic the low glucose/high lactate condition observed in the FCD brain. Cells grown in normal DMEM (0 mM lactate) media were used as controls. The DNA methylation inhibitor, 5-Aza-2′-deoxycytidine (5Aza, decitabine) (Sigma-Aldrich, USA, #A3656), was evaluated at concentrations of 0, 5, 10, and 20 μM for cytotoxicity evaluation [21] (see Methods S1). After 24 h, cells were lysed using RIPA lysis buffer with 1% protease inhibitors for western blot analysis of the target proteins [9]. The antibodies are listed in Table S2.

### Glucose Uptake Assay of [^3^H]-2-Deoxyglucose

EPI-EC and HBMEC were treated for 24 h with either 1 mM low glucose and 20 mM lactate or 5 mM normal glucose and 0 mM lactate, with and without 5Aza (10 μM) at 37 °C. Then cells were washed three times with glucose-free media and a final concentration of 1 μCi/mL [^3^H]-2-deoxyglucose and incubated for 15 min. Cells were then washed twice with ice-cold PBS and solubilized in 1% SDS (modified method [22]) and the radioactivity of the sample with scintillation fluid was determined using a Beckmann Coulter counter. The protein concentration was determined using the Bradford method. Results were expressed as ^3^[H]-2-deoxyglucose uptakes (cpm per μg protein).

### ATPase Activity Assay

The ATPase activity was measured by detecting the free inorganic phosphate (Pi) using a Pi Per Phosphate Assay kit (Molecular Probes #P22061) using HBMEC, EPI-ECs, and/or HEK cell lysates [23]. The standards and samples reacted with Amplex Red reagent for 60 min at 37 °C using a fluorescence microplate reader, at excitation/530–560 nm and emission590 nm. The levels are represented as μmol of Pi per μg of protein in each specimen (details in Methods S1).

### Adenylate Kinase (AK)-Cytotoxicity Assay

Cytotoxicity was assessed in primary ECs (HBMEC and EPI-EC) and HEK by measuring the levels of AK in the supernatant (details in Methods S1).

### Statistical Analysis

Origin Pro 2022 Software was used for data analysis and statistical data interpretation. Student’s *t-*test was used to compare the DNA methylation in FCD-subtypes and matched blood and brain samples. Either one-way or two-way analysis of variance (ANOVA) was utilized to compare multiple groups, with a Tukey post hoc or Bonferroni test for western blot quantification and the glucose-lactate levels. One-way ANOVA was used for ATPase assay values and glucose uptake. All data are shown as mean ± standard error of the mean (SEM). *P-*value of < 0.05 was considered statistically significant.

## Results

### Hypermethylation of GLUT1 and Other Key Genes Responsible for Glucose Metabolism Distinguishes FCDIIa/b from Other FCD Subtypes in the Brain

**Fig. 1:**
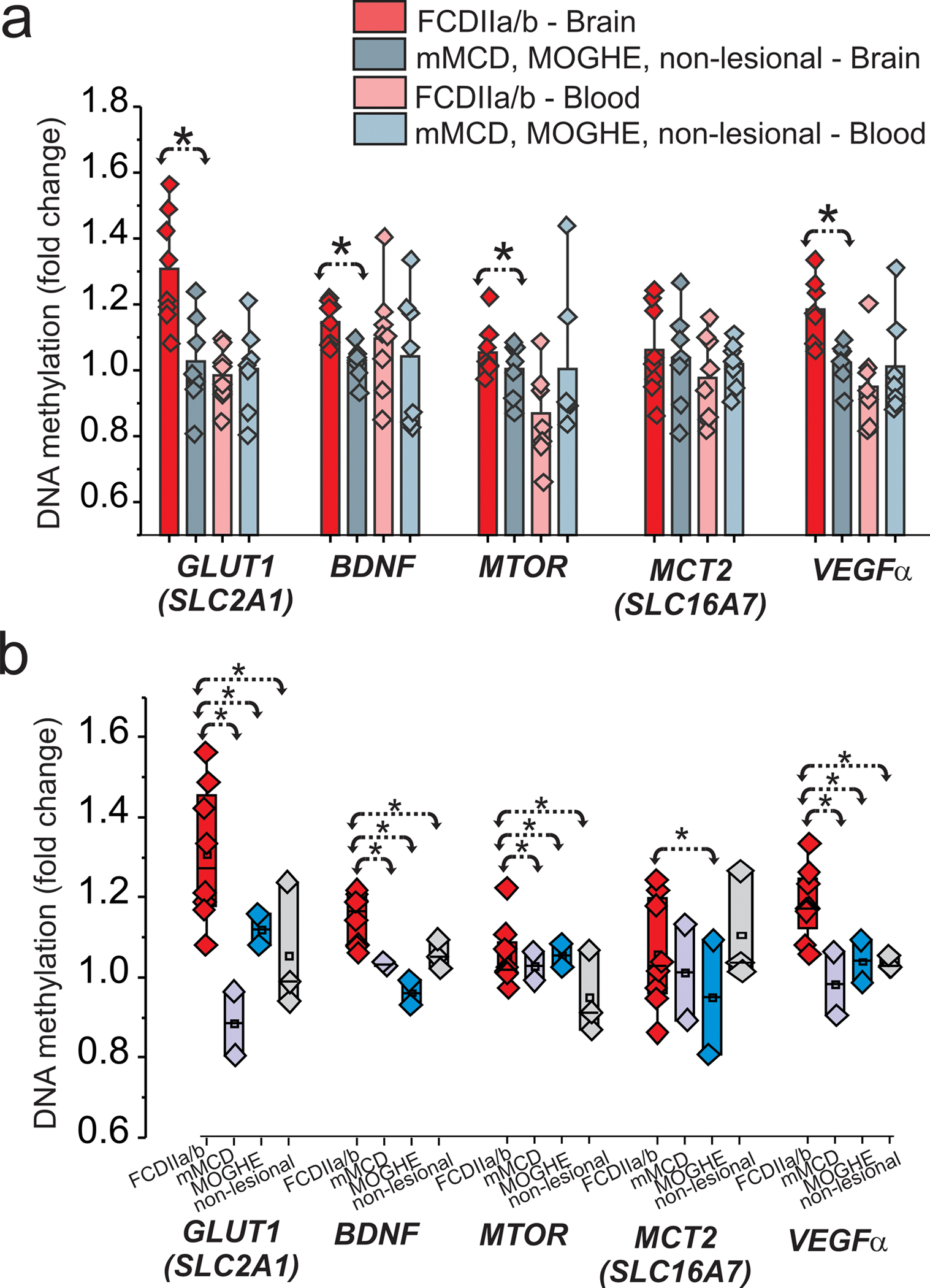
Hypermethylation of GLUT1 and glucose regulatory genes in FCDIIa/b vs. other-FCD subtypes in the brain. GLUT1 (*SLC2A1*), brain-derived neurotrophic factor (*BDNF*), *MTOR*, and MCT2 (*SLC16A7*) brain and matched blood levels (*n* = 15) were compared between FCDIIa/b vs. other-FCD subtypes, i.e., FCDIIa/b vs. mMCD, MOGHE, and non-lesional within the patient cohort (**a**) and within FCD subtypes (**b**) in brain tissues. Fold-change using nominal *p*-value from generalized linear model shows gain of methylation in individuals with FCD subtypes for *SLC2A1*, *BDNF*, and *MTOR* using *t*-test (*p < 0.05) for comparison

Because recent studies have shown the expression of glucose metabolism pathways are regulated by DNA methylation [18], we assessed its role in brain tissues and matching blood samples obtained from FCD subtypes (FCDIIa/b, mMCD, MOGHE) and non-lesional cases. DNA methylation changes were observed in the brain separate FCDIIa/b from other FCD subtypes, mMCD, MOGHE, and non-lesional samples (Fig. 1a). A significant (*p < 0.05) fold-change was found for specific targets in brains related to glucose metabolism, *SLC2A1*, *BDNF*, *MTOR*, and *VEGFα* within FCDIIa/b vs. other FCD subtypes (Fig. 1a). No significant difference was seen in the blood (*n* = 15). We found that DNA methylation of GLUT1 (*SLC2A1*) and other key genes (*BDNF*, *MTOR*, and *VEGFα*) are significantly increased in FCDIIa/b brains (Fig. 1b). This DNA methylation pattern of GLUT1 (*SLC2A1*) and other key genes (*BDNF*, *MTOR*, and *VEGFa*) remained same regardless of the age in these individuals (Fig. S7 ). These target genes showed a marked difference when compared to mMCD, MOGHE, and non-lesional samples (Fig. 1b), thereby categorizing FCDIIa/b from other subtypes using gene methylation as a biomarker.

### Suppressed GLUT1 and Low Glucose-Lactate Ratios Correspond to Elevated VEGFα and MCT2 in FCDIIa and FCDIIb Brain Tissues, Independent of Age and Gender and Not Correlated to Age of Seizure-Onset or Duration of Epilepsy

**Fig. 2:**
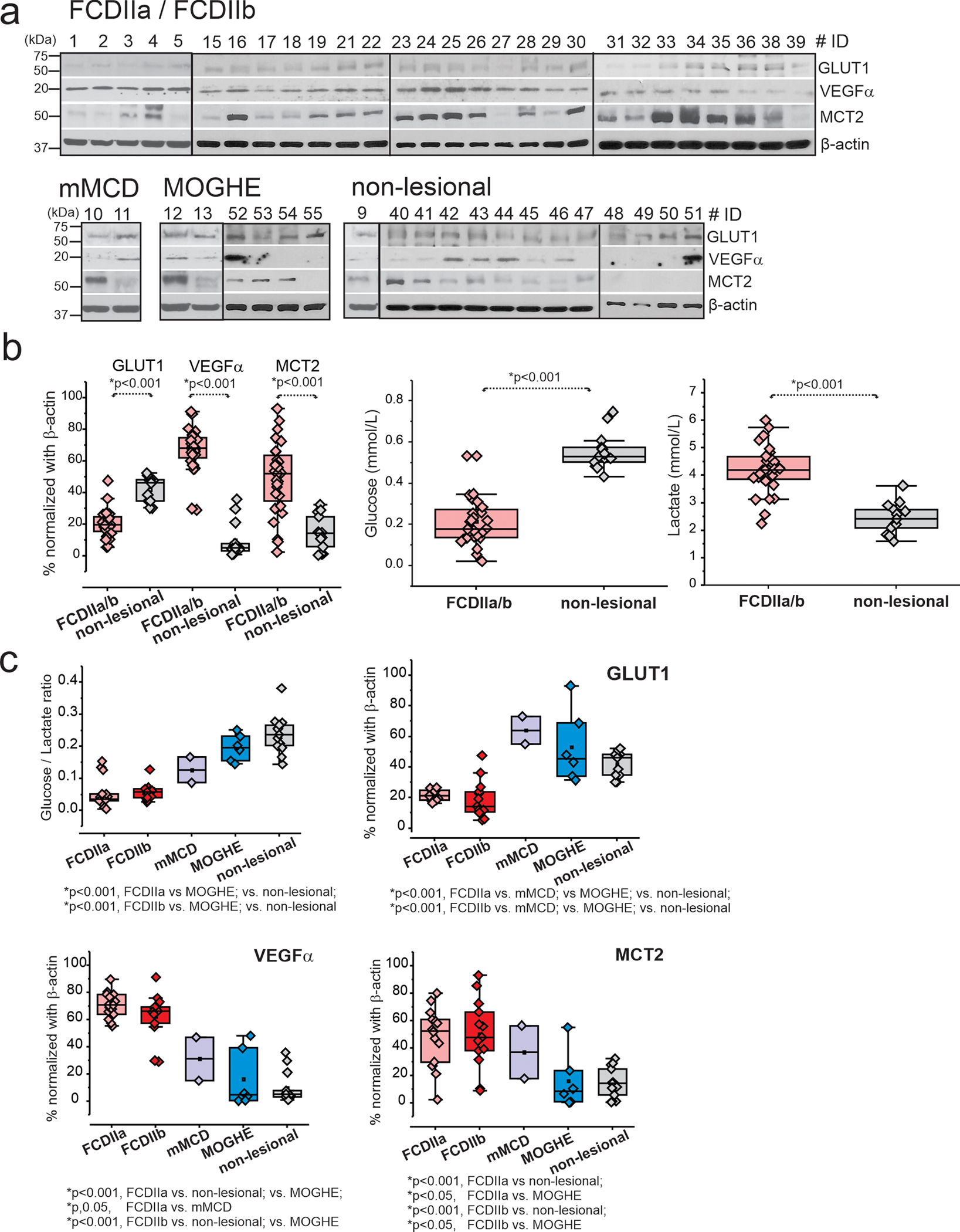
Decreased GLUT1 levels, upregulated VEGFα and MCT2, and low glucose and high lactate levels in FCDIIa/b brain tissues. **a** Western blot shows decreased GLUT1 levels (55 kDa), and upregulated VEGFα (20 kDa) and MCT2 (53 kDa) in FCDIIa/b, mMCD, MOGHE, and non-lesional brain tissues (*n* = 49). β-actin (43 kDa) is used as loading control. **b** The quantified data of western blot is normalized with loading control, β-actin, and plotted to compare FCDIIa/b vs. non-lesional. In addition, the same samples analyzed by biochemical assay showed lower glucose and elevated lactate (in mmol/L). Values are mean ± SEM, ***p < 0.001, one-way ANOVA. **c** Among FCD subtypes, a decreased glucose/lactate ratio and GLUT1 protein levels were significant in FCDIIa and FCDIIb compared to other FCD subtypes, mMCD, MOGHE, and non-lesional. In contrary, increased levels of VEGFα and MCT2 were noted in the respective FCDIIa/b vs. mMCD, MOGHE, and non-lesional. The statistical comparison was done by one-way ANOVA with interaction to compare different FCD subtypes and non-lesional, using a Tukey post hoc test. All data are presented as mean ± SEM, and *p < 0.05 was statistically significant

Western blot analysis performed on 49 samples indicates a marked decrease in GLUT1 expression, coupled with an increase in VEGFα and MCT2 proteins in FCDIIa/b tissues when compared to mMCD, MOGHE, and non-lesional tissue variants, as depicted in Fig. 2 and Fig S8. Notably, glucose concentrations were significantly reduced (*p < 0.001) in FCDIIa/b, whereas lactate levels were found to be higher relative to non-lesional brain tissue. Furthermore, the observed suppression of GLUT1 and the corresponding low glucose-to-lactate ratios were associated with increased VEGFα levels in FCDIIa/b, as shown in Fig. 2b, c.

An analysis to assess the potential correlation between age or gender and the protein expression in FCDIIa/b versus non-lesional samples was performed. This comparison, illustrated in Figs. S1 and S2, spanned various age brackets (0–20; 21–45; > 45 years). The results demonstrated no significant differences across age or gender subgroups, with the reduction in GLUT1 and the rise in VEGFα and MCT2 levels in FCDIIa/b remaining consistent across all categories. Similarly, the glucose-to-lactate ratio was consistently lower in FCDIIa/b brain tissues across these age groups compared to non-lesional tissues, as shown in Fig. S1.

Further examination revealed that the alterations in glucose-to-lactate ratios and the levels of GLUT1, VEGFα, and MCT2 proteins in FCDIIa/b versus non-lesional tissues were not influenced by gender (Fig. S2), age of seizure onset (Fig. S3), or the duration of epilepsy (Fig. S4) in either FCDIIa/b or non-lesional subjects. Collectively, these findings underscore a distinct glucose metabolic profile associated with disease pathology and FCD subtype.

### Increased mTOR Signaling in FCDIIa/b Brains

**Fig. 3:**
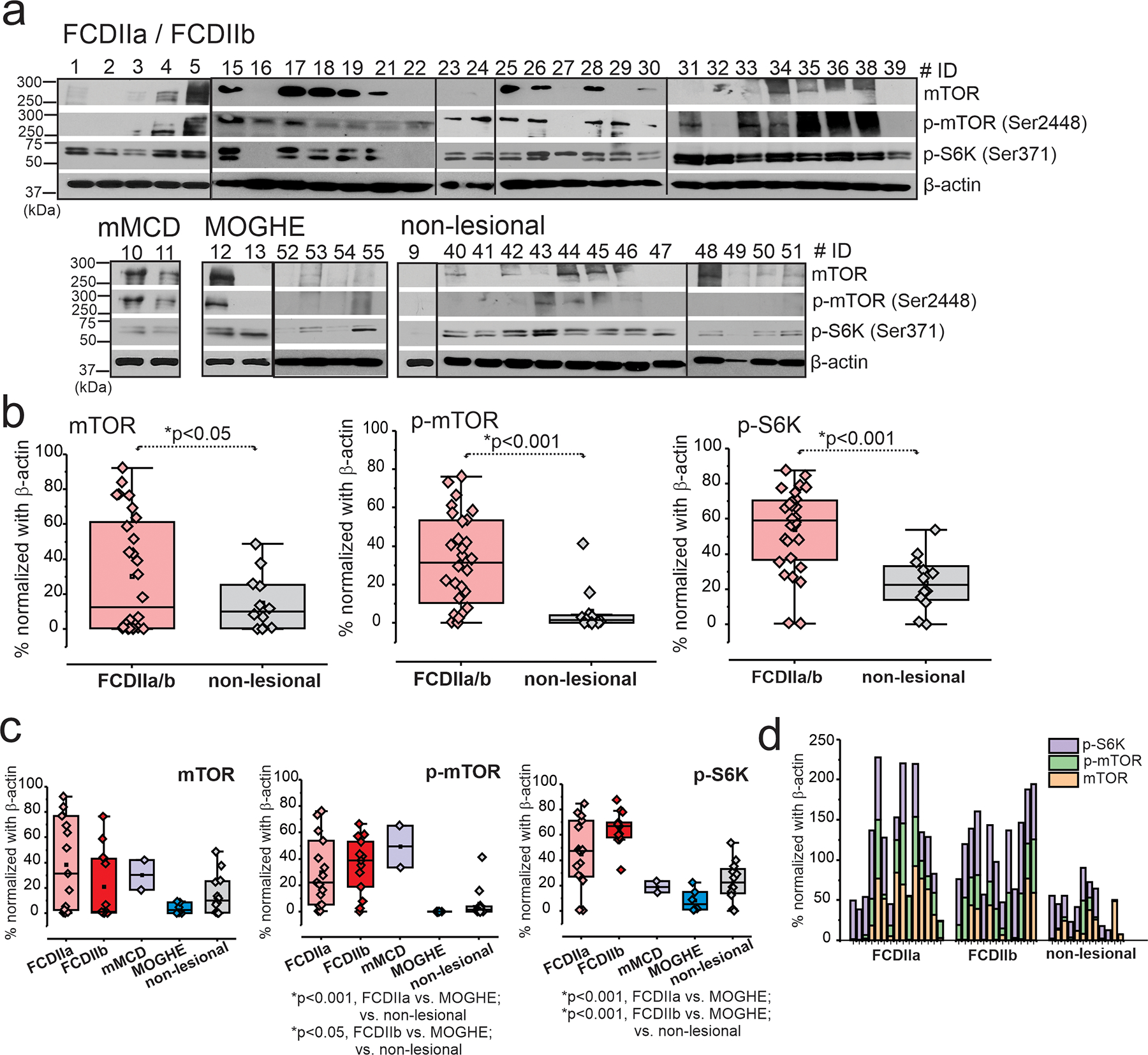
Overall increased mTOR signaling pathway is predominant in dysplastic brains. **a** In the FCDIIa/b brain tissues, elevated levels of mTOR (289 kDa), p-mTOR (Ser2448) (289 kDa), and p-S6K (Ser371) (70 kDa) by western blot analysis are evident compared to non-lesional, shown by the representative blot (*n* = 49). **b** The values of these targets analyzed by western blot were normalized with β-actin (43 kDa) and plotted that shows significant increase in mTOR (*p < 0.001), p-mTOR (*p < 0.001), and p-S6K (*p < 0.001). Values are mean ± SEM, ***p < 0.001, two-sample *t*-test for comparison between FCD vs. non-lesional brain tissues. Among FCD subtypes, FCDIIa and FCDIIb showed relatively increased mTOR activation compared to other FCD subtypes, mMCD, MOGHE, and non-lesional where elevation in mTOR activated, p-mTOR (*p < 0.001, FCDIIa vs. non-lesional; *p < 0.05, FCDIIa vs. MOGHE; *p < 0.05, FCDIIb vs. MOGHE/vs. non-lesional), and p-S6K (*p < 0.001, FCDIIa vs. MOGHE; *p < 0.001, FCDIIb vs. MOGHE/non-lesional) was observed by western blot analysis normalized with β-actin. Pathology comparison in individual levels shows similar trend of increased elevation of mTOR, p-mTOR, and p-S6K in FCDIIa and FCDIIb compared to non-lesional. Values are mean ± SEM, ***p < 0.001, one-way ANOVA post hoc Tukey test used for comparison within groups

Cortical brain tissues derived from patients with different FCD subtypes revealed significant (*p < 0.05) mTOR overexpression (Fig. 3b) and activated mTOR pathway in FCDIIa/b vs. MOGHE and non-lesional (Fig. 3c and Fig. S7). However, no significant difference was observed between FCDIIa/b (*n* = 28) and mMCD (*n* = 2). We observed significant elevation of *p*-mTOR (*p < 0.001) and p-S6K (*p < 0.001) in FCDIIa/b vs. non-lesional brain tissues (Fig. 3 and Fig. S9) and statistically significant increased levels within groups (Fig. 3a–c). Furthermore, the individual comparison of subjects (Fig. 3c, d) clearly distinguishes the level of mTOR, p-mTOR, and p-S6K of FCDIIa (*n* = 15) and FCDIIb (*n* = 13) from MOGHE (*n* = 6) and non-lesional (*n* = 12).

### Pharmacological Inhibition of DNA Methylation Increased GLUT1 Levels and Decreased VEGFα in EPI-ECs with No Cellular Stress or Cytotoxicity

**Fig. 4:**
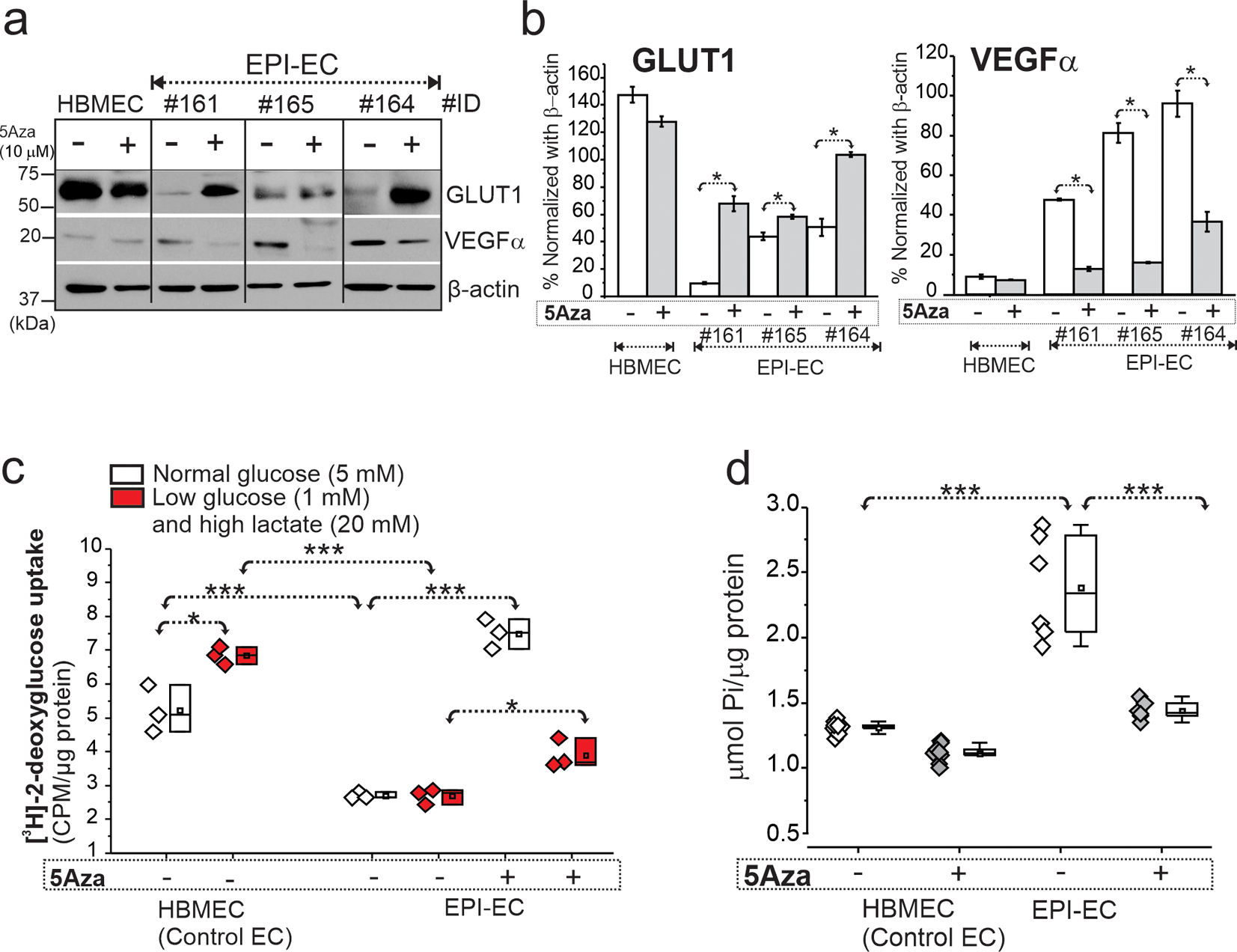
Restoration of GLUT1 and VEGFα levels, glucose uptake function and ATPase activity in FCD EPI-ECs post-DNA methylation inhibition. **a**–**b** DNA methylation inhibitor 5Aza (decitabine) treatment significantly increased GLUT1 levels (*p < 0.05) and decreased VEGFα (*p < 0.05) in EPI-ECs compared to untreated cells counterpart. Individual EPI-EC from respective patients were denoted by ID #161, #165, and #164 and compared with normal EC (HBMEC). Values are mean ± SEM, *p < 0.05, two-sample *t*-test for comparison between conditions, with and without 5Aza treatment. **c** Glucose uptake assessed by [^3^H]-2-deoxyglucose radiotracer showed increased uptake in HBMEC under low glucose-high lactate condition compared to normal glucose treatment. However, EPI-EC, under both normal and low glucose-high lactate conditions, showed non-significant difference. Furthermore, with 5Aza (10 μM) co-treatment in EPI-ECs, the glucose update pattern in cells both under normal (*p < 0.001) and low glucose-high lactate (*p < 0.05) conditions increased significantly compared to respective untreated 5Aza EPI-EC. Values are mean ± SEM, ***p < 0.001, one-way ANOVA with post hoc Tukey test used for group comparisons. **d** The increased (*p < 0.001) ATPase activity is noted in the EPI-EC compared to HBMEC, which is found to be significantly decreased (*p < 0.001) with 5Aza pre-treatment to a level more comparable to control EC. Values are mean ± SEM, ***p < 0.001, one-way ANOVA with post hoc Tukey test used for group comparisons

DNA methylation inhibition with decitabine (5Aza) increased GLUT1 levels (*p < 0.05) and decreased VEGFα (*p < 0.05) in FCD EPI-EC compared to control human brain cortical microvascular endothelial cells (HBMEC) which was plotted as percentage normalized with β-actin (Fig. 4a, b, Fig. S10). There was no cellular stress or cytotoxicity observed in either primary brain ECs or HEK treated with 5, 10, and 20 μM 5Aza (decitabine) when evaluated after 2–24 h (Fig. S5, Fig. S6) (see “Materials and Methods” section and Table S1 for EPI-EC details).

### Decitabine Regulates Glucose Metabolic Functional Activity in EPI-EC and HEK

In control HBMEC, we observed increased [^3^H]-2-deoxy-glucose uptake under low glucose-high lactate (*p < 0.05) when compared to normoglycemic conditions. In EPI-EC, [^3^H]-2-deoxyglucose uptake was impaired under physiological and low glucose-high lactate condition compared to HBMEC (*p < 0.001). Decitabine restored glucose uptake function of [^3^H]-2-deoxyglucose of EPI-EC under normal glucose (*p < 0.001) and low glucose-high lactate (*p < 0.05) condition compared to untreated EPI-EC, suggesting DNA methylation inhibition regulates the cellular response from dysfunctional glucose uptake observed in EPI-ECs (Fig. 4c). ATPase overactivity in EPI-EC vs. HBMEC (*p < 0.001) is also retained back to normal levels (*p < 0.001), post-decitabine stimulation (Fig. 4d). These findings suggest gene methylation regulates glucose-uptake and ATPase activity in EPI-ECs.

**Fig. 5:**
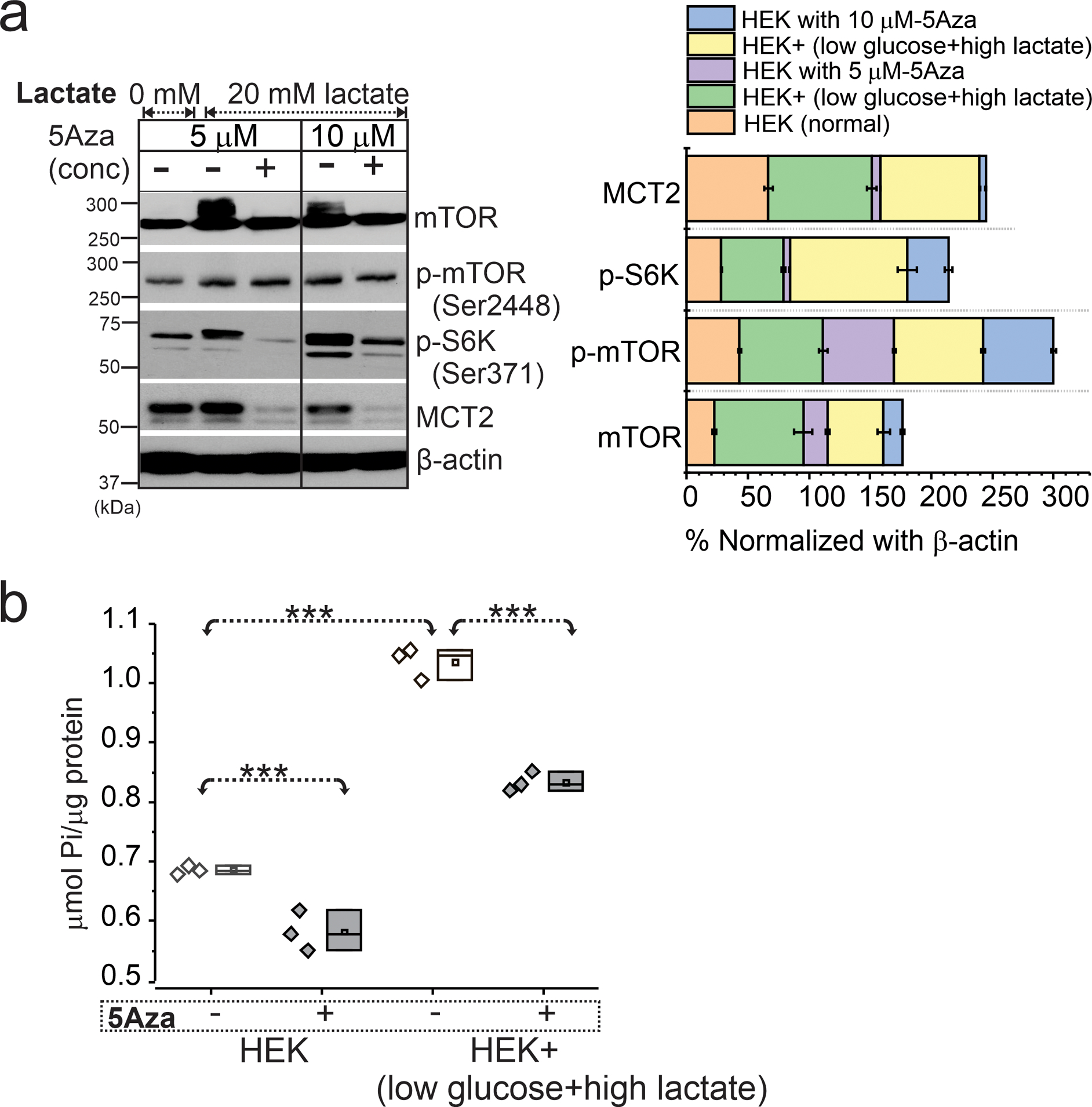
Upregulated mTOR and ATPase activity on HEK cells with low glucose-high lactate state are modulated by DNA methylation. **a** HEK cells treated with low glucose-high lactate increased mTOR, p-mTOR, p-S6K, and MCT2 levels at 24 h. Inhibition of DNA methylation with 5Aza (decitabine) at both 5 and 10 μM reduced mTOR activation and MCT2 levels significantly. The overall trend under different conditions, with and without 5Aza, is plotted after normalization with β-actin. **b** Upregulated ATPase activity in low glucose-high lactate (*p < 0.001) was evident compared to normal (glucose and low lactate state). Further pretreatment of 24 h with 5Aza significantly decreased (*p < 0.001) ATPase activity in both these conditions, i.e., low glucose-high lactate and normal glucose treated cells. Values are mean ± SEM, ***p < 0.001, one-way ANOVA with post hoc Tukey test used for group comparisons

Similarly, HEK cells known to exhibit several properties of immature neuronal cells when stimulated by hypometabolic condition (Fig. 5a, b and Fig. S11) showed upregulated mTOR activity while decitabine significantly reduced mTOR signaling and MCT2 levels. ATPase overactivity in the same HEK cells was evident in low glucose-high lactate condition, compared to normal glucose condition as the hypometabolic-state was maintained. However, such energy demand was restored back to normal ATPase activity (Fig. 5b) in HEK + decitabine group compared to untreated cells, suggesting a role of DNA methylation in regulating mTOR signaling and ATPase in the cells.

## Discussion

The role of DNA methylation on GLUT1 and hypometabolism in focal cortical dysplasia (FCD) remains poorly understood. In this study, we show a differential methylation of key genes involved with glucose uptake and metabolism in epileptic FCDIIa/b. We also compared the influence of methylation on GLUT1 and glucose regulation in FCDIIa/b versus mMCD, MOGHE and non-lesional brain tissues. We further show that pharmacological inhibition of DNA methylation increases GLUT1 levels, restores cellular glucose uptake function, and reduces ATPase activity and mTOR pathway activation. These results collectively suggest (1) glucose uptake reduction is possibly due to GLUT1 suppression, (2) hypermethylation of *SLC2A1*/GLUT1 and other glucose regulatory genes are specific to FCDIIa/b, and (3) cellular and functional links exist between hypermethylation and GLUT1 downregulation.

Recent studies have shown an integrated pathological and molecular classification of FCD subtypes based on genetic and epigenetic determinants in focal epilepsies [24, 25]. Among the 15 patients in our study with no FCD on histopathology, six were seizure-free post-surgery, suggesting that the resected tissue indeed contributed to the epileptogenic zone. It is important to note that the primary goal of this analysis was not to compare epileptic tissue to non-epileptic tissue, but rather to examine tissue with well-categorized FCD pathology in comparison to tissue with no FCD on histopathology. In the context of cerebral hypometabolism in this study, we have identified that DNA hypermethylation of GLUT1 (*SLC2A1*) and key glucose regulatory genes, including *MTOR*, *BDNF*, *VEGFα*, and MCT2 (*SLC16A7*), distinguishes FCDIIa/b from other FCD-subtypes (mMCD, MOGHE) and non-lesional in brain samples. The relevance of hypermethylation of GLUT1 and other glucose regulators as potential biomarkers are indicators of the downstream molecular mechanism unfolding, advancing the clinicopathological understanding in FCD [16, 25, 26]. Other key transcription factors, non-coding RNAs, or other epigenetic regulators may be involved in modulating the expression of genes such as *SLC2A1* (GLUT1), *VEGFα*, and related genes. Chromatin factor high-mobility group A1 (HMGA1) was found to play a key role in the transcriptional regulation of glucose responsive genes, including some that were involved in forkhead transcription factor (FoxO1)-mediated glucose metabolism and FoxO1 was found to be reduced in subjects carrying defects in HMGA1 [27]. Literature also suggests that GLUT-1 expression is regulated by microRNAs affecting other gene targets observed in glioblastoma and other human tumors [28]. One study found that hypoxia-induced miR-153 regulated the HIF1α/VEGFα axis in breast cancer angiogenesis and proposed an opportunity of miR-153 as an anti-angiogenesis therapy [29]. In addition, hypoxia-regulated expression of GLUT1 identified at different stages of glioblastoma oncogenesis, as tumor cells and macrophages under hypoxic conditions migrated to the foci of necrosis secrete factors that stimulate angiogenesis, including VEGF, platelet-derive growth factor, transforming growth factor β, epidermal growth factor, tumor necrosis factor-alpha, and activation of matrix metalloproteinases [28]. Deletion of enhancers and long non-coding RNA (lncRNAs) in another report was found to promote significant changes in *VEGFA* and *VEGFC* expression suggesting a functional role of these lncRNA in angiogenesis affecting endothelial cell function [30]. Such reports collectively elucidate that DNA methylation and epigenetic modifications could play a significant role in influencing the expression of GLUT1 (*SLC2A1*), *VEGF*, and related glucose regulatory genes.

The key cell types (e.g., endothelial, astroglial, and neuronal cells), majorly involved in brain parenchymal glucose transport across the blood–brain barrier (BBB), astrocyte-neuronal lactate shuttle, and metabolism, contribute to epileptogenesis and seizure propagation [8]. Beside genomic DNA methylation differences between human FCD subtypes, our findings also demonstrated GLUT1 suppression and increased glucose metabolism with low-glucose and high brain lactate levels corresponded to elevated VEGFα in FCDIIa and FCDIIb brain tissues proteins supporting a role of endothelial and astroglial cells in the pathophysiology. This is suggestive of a possible effect of methylation on glucose metabolism affecting glucose transport, via GLUT1, and altered glucose to lactate conversion, beside a possible influence on angiogenesis as seen by VEGFα upregulation.

Suppression of GLUT1 in cortical dysplasia could contribute to the interictal hypometabolism prevalent in epilepsy [5, 8, 10]. Such low cerebral glucose levels have also been reported post-severe traumatic brain injury [31, 32]. FCDIIa and FCDIIb are known to be the most common malformations of cortical development among children with drug-resistant focal epilepsy [16, 33, 34]. The findings in this study suggest GLUT1 and glucose metabolism remains significantly altered in FCDIIa and FCDIIb compared to non-lesional tissue samples across various age brackets (0–20, 21–45, and over 45 years old). The suppression of GLUT1 and low glucose-lactate ratio with elevated VEGFα and MCT2 in FCDIIa/b vs. non-lesional cases is not age and gender dependent, thereby confirming the metabolic changes observed are specifically due to disease pathology. Despite the trend of GLUT1 suppression and low glucose-lactate ratio and higher VEGFα and MCT2 in FCDIIa/b, we observed no correlation with age of seizure onset or the duration of epilepsy. Such hypometabolic signatures could be valuable for diagnosis regardless of factors such as individual age, gender, age of seizure onset, or duration of epilepsy.

Previous reports have shown high-spiking and low-spiking tissues with altered metabolic patterns in human epileptic brain [35]. Low GLUT1 in FCD and glucose deprivation could also predispose neurons to synchronous firing and seizure generation as reported by others [10, 36]. Indeed, altered molecular function in human dysplastic versus non-dysplastic brain regions was also recently reported [19]. Protein translation is further enhanced by mTORC1 signaling via phosphorylation of S6 Kinase (p-S6K) to activate the ribosomal protein S6, a component of the 40S ribosomal subunit. Therefore, our observations of mTOR cascade activation in FCD are consistent with previous reports that indicate enhanced mTOR activation in hemimegalencephaly [37, 38] and ganglioglioma [39] pathologies and distinguish tubers from FCD [40, 41]. Herein, the p70S6K and p-S6 isoforms in resected FCDIIb specimens, indicating phosphorylated molecules activating downstream targets of the signaling pathways. These reports summarize the molecular events leading to abnormal brain development resulting in mTOR activation evidenced by hyperphosphorylation of mTOR, p-S6K, and S6 proteins. Despite individual variability, we observe significant mTOR activation, particularly the upregulation of p-mTOR and p-S6K in FCDIIa, FCDIIb across subjects compared to non-lesional.

To validate the consequence of these epigenetic changes, the decreased GLUT1 and increased VEGFα levels in FCD EPI-ECs were reversed using the DNA methylation inhibitor, decitabine. Furthermore, glucose uptake dysfunction in EPI-ECs was also rescued within both normal glucose and low-glucose with high-lactate conditions, suggesting a beneficial effect of decitabine on the EPI-ECs, and supporting a role for DNA methylation in regulating glucose trafficking (GLUT1) and angiogenesis (VEGFα) at the BBB. It is also reported that BBB disruption influenced the DNA methylation events via increased expression of noncoding RNA miRNA29b which affects the expression of DNA methyltransferase enzyme (DNMT3b) and matrix metalloproteinases (MMP9). Furthermore, decitabine ameliorates the BBB damage by reducing the expression of miRNA29b [42]. These findings are collectively of great relevance as compromised BBB endothelial function and epilepsy with cortical dysplasia are observed [5, 9, 19, 43]. Future studies could elucidate how BBB leakage-induced stress contributes to DNA methylation at the neurovascular unit. These findings corroborate the mechanism of brain metabolic disturbance via DNA methylation which could pave the way toward potential early intervention strategy in FCD.

Genetics and environmental factors are associated with complex neurodevelopmental disorders [44, 45]. Factors such as inflammation, potential drug treatment, dietary, and lifestyle choices or genetic predisposition of individuals such as inheritable mutations may influence DNA methylation in FCD subtypes that could be considered in future studies. Developmental or early neuroinflammatory exposure has shown to impact the cerebral methylation with wide-spread epigenetic modifications especially in neurodevelopment related genes studied in rodents [46]. Medications, for example, sodium valproate, a histone deacetylating inhibitor, have shown in studies to modulate DNA methylation via activating TET2 genes and protein overexpression in both G1-phase arrested and proliferative cells [12], which could be further examined. It is also noteworthy that genetic inheritance, for example, *Glut1* deficiency syndrome, a rare metabolic encephalopathy, was associated with abnormal brain metabolism, characterized by seizures appearing early in life and often drug resistant, movement disorders, and intellectual disability [47, 48]. Evidence also suggests both ketogenic diet [49–51] and developmental energy restriction [52] could modulate brain glucose transporter expression and neurodevelopment. Of importance, ketogenic diet demonstrates as a promising disease-modifying or partial antiepileptogenesis therapy for epilepsy and has revealed an epigenetic mechanism for increase the adenosine level in the brain and inhibition of DNA methylation [53, 54]. Dysregulation of leptin and insulin signaling pathways within brain regions may contribute not only to the development of obesity, but also systemically affect the peripheral organs, thereby manifesting as metabolic diseases involving altered DNA methylation [55]. Together, such environmental and genetic factors may contribute to the methylation changes underlying FCD pathology subtypes.

Signaling of mTOR, p-mTOR (Ser2448), and p-S6K (Ser371) by decitabine was observed in HEK cells under low glucose-high lactate condition. The elevated MCT2 levels decreased following decitabine stimulation suggesting a role for gene hypermethylation in regulating MCT2 levels and mTOR signaling. Lactate is also known to activate the mTORC1 complex in cancer cells [56], and the activation of mTOR signals via HIF1α initiates VEGF expression in FCDIIb [57]. Higher ATPase activity in cells is similarly downregulated to control levels with decreased phosphate activity following decitabine stimulation. Studies have also reported that lactates operate via negative feedback on neuronal activity by a receptor-mediated mechanism, independent from its intracellular metabolism [58]. Moreover, decitabine was previously shown in fully kindled rat model to increase pentylenetetrazole (a GABA receptor antagonist)-induced seizure thresholds to attenuate seizures, and to suppress epileptogenesis [54, 59].

## Conclusions

This study highlights a role for DNA hypermethylation on GLUT1 suppression and glucose regulation, which is elevated in human FCDIIa/b subtype as an epigenetic signature related to glucose metabolism. Furthermore, we show DNA methylation activates key glucose regulatory pathway protein targets and cellular function linked to glucose uptake, ATPase activity, and thereby improving functional efficacy. Several potential limitations of the study should be considered. This is a single center study with a relatively small sample size especially while examining many subtypes of FCD and obtaining larger FCD subtypes, sample sizes will be important. Additionally, variability in experimental conditions may introduce confounding factors. Despite these limitations, this study creates an opportunity for more extensive investigation of these glucose-regulatory target biomarkers in boarder FCD patient population. Also, confirming the role of DNA methylation in seizure behavior using decitabine in vivo using a rodent CD model will be critical because it will provide direct evidence linking epigenetic modifications to neuronal activity and seizure susceptibility. Decitabine, a DNA methylation inhibitor, can help establish causation by reversing hypermethylation and assessing its impact on seizure frequency and severity. Although beyond the scope of this study, such insight in the future could pave the way for targeted epigenetic therapies for epilepsy and related neurological disorders. Collectively these findings pave a foundation for clinical intervention and diagnostic strategies of biomarker in FCD related to GLUT1 and hypometabolism anomalies.

## Supplementary Material

**Supplementary Information** The online version contains supplementary material available at https://doi.org/10.1007/s12035-025-04871-z.

Supplemental Fig 8

Supplemental Fig 9

Supplemental Fig 10

Supplemental Fig 11

Supplemental Fig 1

Supplemental Fig 2

Supplemental Fig 3

Supplemental Fig 4

Supplemental Fig 5

Supplemental Fig 6

Supplemental Fig 7

Supplemental Table 1

Supplemental Table 2

Supplemental Method

## Data Availability

No datasets were generated or analysed during the current study.
