# Supplementary material for: GLUT1 and Cerebral Glucose Hypometabolism in Human Focal Cortical Dysplasia Is Associated with Hypermethylation of Key Glucose Regulatory Genes
Source: Mol Neurobiol. 2025 Apr 7;62(8):10264–76. doi: 10.1007/s12035-025-04871-z (PMC12289428; doi:10.1007/s12035-025-04871-z)
Supplement: Supplementary file 1 — Supplementary file1 (DOCX 16 KB) [file 12035_2025_4871_MOESM1_ESM.docx]

**Additional Material and Methods**

**Tissue Lysate Preparation and Western Blot**

Approximately 50 mg of fresh-frozen human cortical tissue resected from patients with drug-resistant epilepsy due to FCD (*n*=49) was lysed with radioimmunoprecipitation assay buffer (RIPA; Sigma-Aldrich, Burlington, MA, USA, catalog number R0278) combined with 1x protease inhibitor cocktail (Sigma, catalog number P8340) as previously described. The protein concentration of the lysates was estimated by the Bradford method.

For the human brain tissue lysates, GLUT1 (55 kDa), VEGFα (20 kDa), MCT2 (53 kDa), mTOR (289 kDa), phospho-mTOR (Ser2448) (289 kDa), phospho-S6K (Ser371) (70 kDa) were separated by 8% or 12% sodium dodecyl sulfate polyacrylamide gel electrophoresis (SDS-PAGE) and transferred to polyvinylidene fluoride (PVDF) membranes (EMD Millipore Corp., Burlington, MA, USA, catalog number IPVH00010) by semi-dry transfer (Trans-Blot© SD Semi-Dry Transfer Cell, Bio-Rad, Hercules, CA, USA). In brief, the membranes were blocked in 5% milk for 2-4 h and later probed overnight at 4°C with the respective primary antibody followed by the appropriate secondary antibody for 1h at room temperature (Table S2). Between probing target proteins, the PVDF membranes were incubated in stripping buffer (ThermoFisher Scientific, catalog number 21059) for 30 min at room temperature followed by blocking of the membranes in 5% milk for 2-4 h before re-probing. In each case, the protein expression was normalized by β-actin (43 kDa) in total lysates as loading controls, and the densitometry quantification of the images was performed using ImageJ software (NIH, Bethesda, MD, USA).

**Glucose-Lactate Measurement**

Glucose and lactate were measured via a dual-channel immobilized oxidase enzyme analyzer (YSI 2700 SELECT; YSI Inc., Yellow Springs, OH) in human brain tissue samples. The glucose and lactate standards were used for calibration and validation of the protocol as previously described. The detection ranges for D-glucose and L-lactate are 0–50 mmol/L and 0–29.98 mmol/L, respectively. D-glucose was calibrated to 13.89 mmol/L and L-lactate to 5.61 mmol/L. Glucose and lactate each have a coefficient of variation (CV) of 2% and a recovery of 97–100%. In brief, 50 mg of frozen brain tissue sample was homogenized in 300 µL of RIPA buffer with protease inhibitor cocktail (Sigma, St. Louis, MO). The tissue lysates were subsequently centrifuged for 30 min at 4°C, and the sample supernatants were analyzed for the glucose and lactate levels measured in mmol/liter, and the data plotted as glucose/lactate ratio.

**Primary Brain Endothelial Cell Culture and Human Embryonic Kidney Cells**

Briefly, resected epileptic brain tissue specimens (Table S1) were incubated in collagenase type II (2 mg/mL: ThermoFisher Scientific, catalog number 17101-015) at 37°C for 40 min to dissociate the ECs. The collagenase was then washed with endothelial cell medium (1.5 g/100 mL MCDB-105, Sigma-Aldrich, catalog number M6395), 9-10 mg/100 mL endothelial cell growth supplement (EMD Millipore, catalog number 02-102), 800 U/100 mL heparin (Sigma-Aldrich, catalog number H3149), 10% fetal bovine serum (Atlas Biologicals, Fort Collins, CO, USA, catalog number F-0500-DR), and 1% penicillin/streptomycin), and the dissociated cells were plated initially in fibronectin-coated (Sigma-Aldrich, catalog number F4759; 3 µg/cm^2^) 75 cm^2^ tissue culture flasks. Primary control human brain microvascular endothelial cells (HBMECs) were purchased from Cell Systems (Kirkland, WA, USA, catalog number ACBRI 376). The HBMECs were used as a control compared to the EPI-EC. According to the company, the HBMECs were dissociated from normal human brain cortical tissue obtained from healthy donors using a Beckman elutriation system and characterized by von Willebrand factor staining. Other specific details are available on the company website Cell Systems, [Primary Human Brain Microvascular Endothelial Cells (ACBRI 376) – Cell Systems (cell-systems.com)](https://cell-systems.com/products/human-brain-microvascular-endothelial-cells-acbri-376?variant=37945739019).

**ATPase activity assay**

The ATPase activity was measured by detecting the free inorganic phosphate (Pi) using a Pi Per Phosphate Assay kit (Molecular Probes, catalog number P22061) using the cell lysates (HBMEC and FCD EPI-ECs or HEK). The assay measures an increase in fluorescence absorption of an Amplex Red reagent that is proportional to the amount of Pi in the samples. Briefly, a standard curve was obtained by using different known concentrations of Pi. The standards and samples reacted with Amplex Red reagent for 60 min at 37°C in the dark using a fluorescence microplate reader (BioTek, Synergy HT, USA), set for excitation at 530–560 nm and emission detection at 590 nm. The levels are represented as μmol of Pi per μg of protein in each specimen.

**Adenylate Kinase- cytotoxicity assay**

This highly sensitive assay measures adenylate kinase (catalog number LT07-217; Lonza) released from damaged mammalian cells and provides an accurate determination of the degree of cytolysis. Adenylate kinase measurements are plotted as relative luminescent units, so that equal numbers of cells (6 × 10^5^ cells/per chamber) exposed to different treatments can be compared with the control and the 0 h time-point. Cytotoxicity measurements were made with/ without 5Aza treated with 5, 10 and 20 μM for 24 h.
