# Supplementary figures and images for: GLUT1 and Cerebral Glucose Hypometabolism in Human Focal Cortical Dysplasia Is Associated with Hypermethylation of Key Glucose Regulatory Genes
Source: Mol Neurobiol. 2025 Apr 7;62(8):10264–76. doi: 10.1007/s12035-025-04871-z (PMC12289428; doi:10.1007/s12035-025-04871-z)

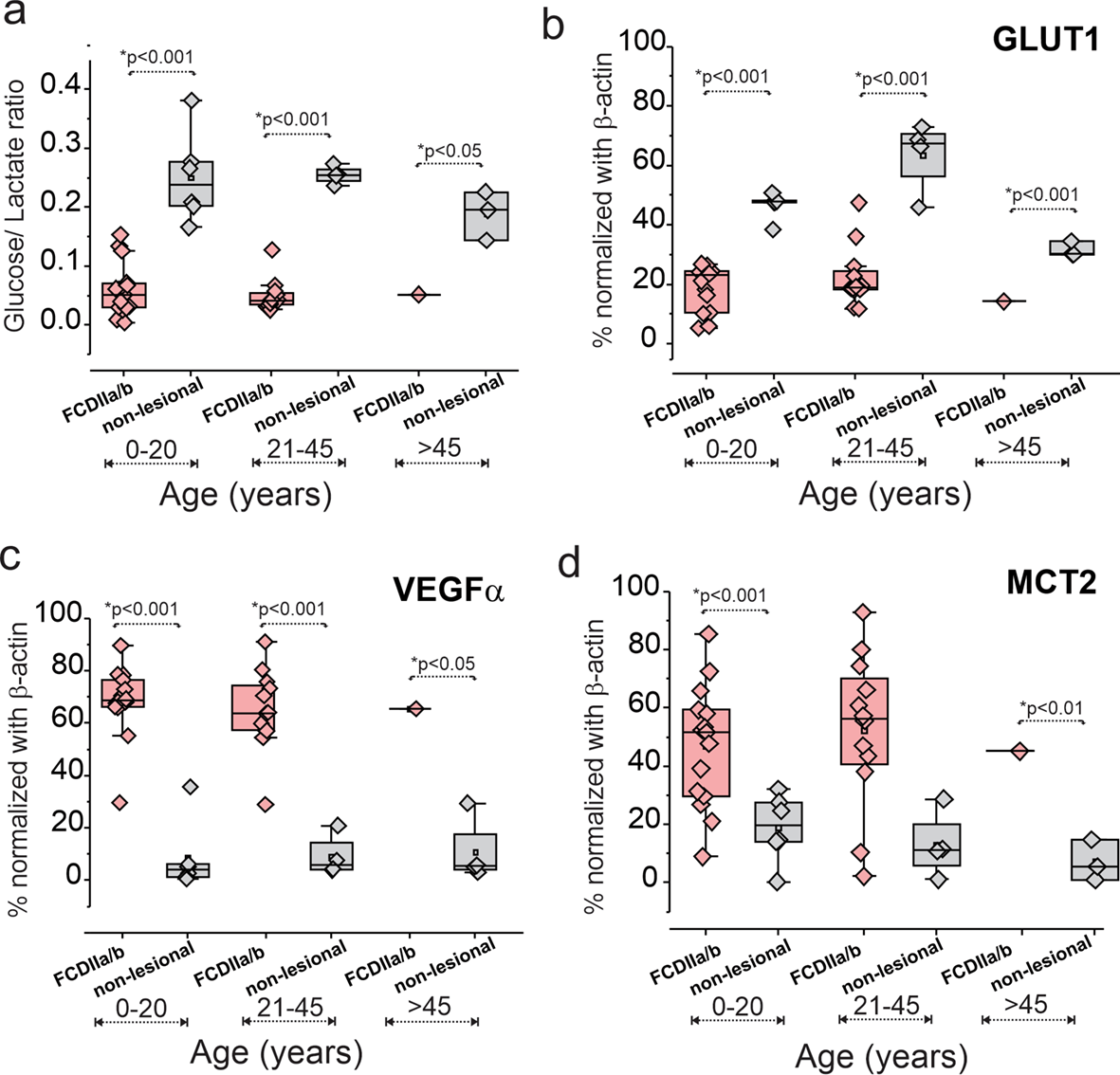

Supplement: Supplementary file 2 — GLUT1 suppression and low glucose/lactate ratios correspond to VEGFα elevation in FCDIIa and FCDIIb brain tissues is independent of age. Across age groups (0-20; 21-45 and above 45 years old) in FCDIIa/b vs. non-lesional (a-d) Decreased brain glucose-lactate ratio by biochemical analysis (a), suppressed GLUT1 levels, and upregulated VEGFα by western blot was observed within ages 0-20 yrs old in FCDIIa/b (n=16) compared to non-lesional (n=11); ages: 21-45 yrs. old in FCDIIa/b (n=14) compared to non-lesional (n=7) and in ages of 45 yrs. or older in FCD (n=1) compared to non-lesional (n=3) group. The quantified data of western blot normalized with loading control; β-actin is depicted (b-d). Values are mean ± SEM, ***p<0.001, two sample t-test. (PNG 302 KB) [file 12035_2025_4871_Fig6_ESM.png]

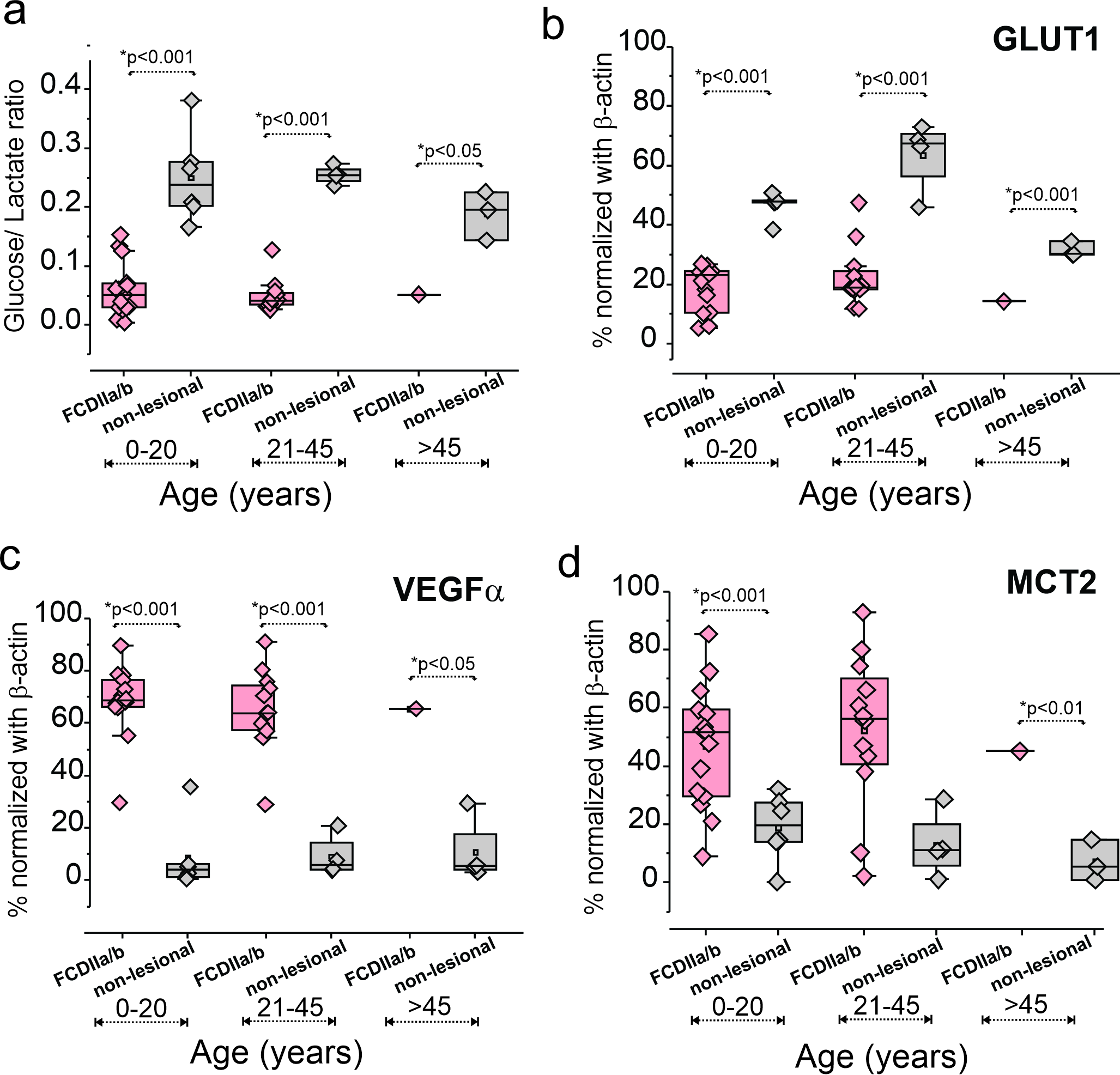

Supplement: Supplementary file 3 — High resolution image (TIF 16.0 MB) [file 12035_2025_4871_MOESM2_ESM.tif]

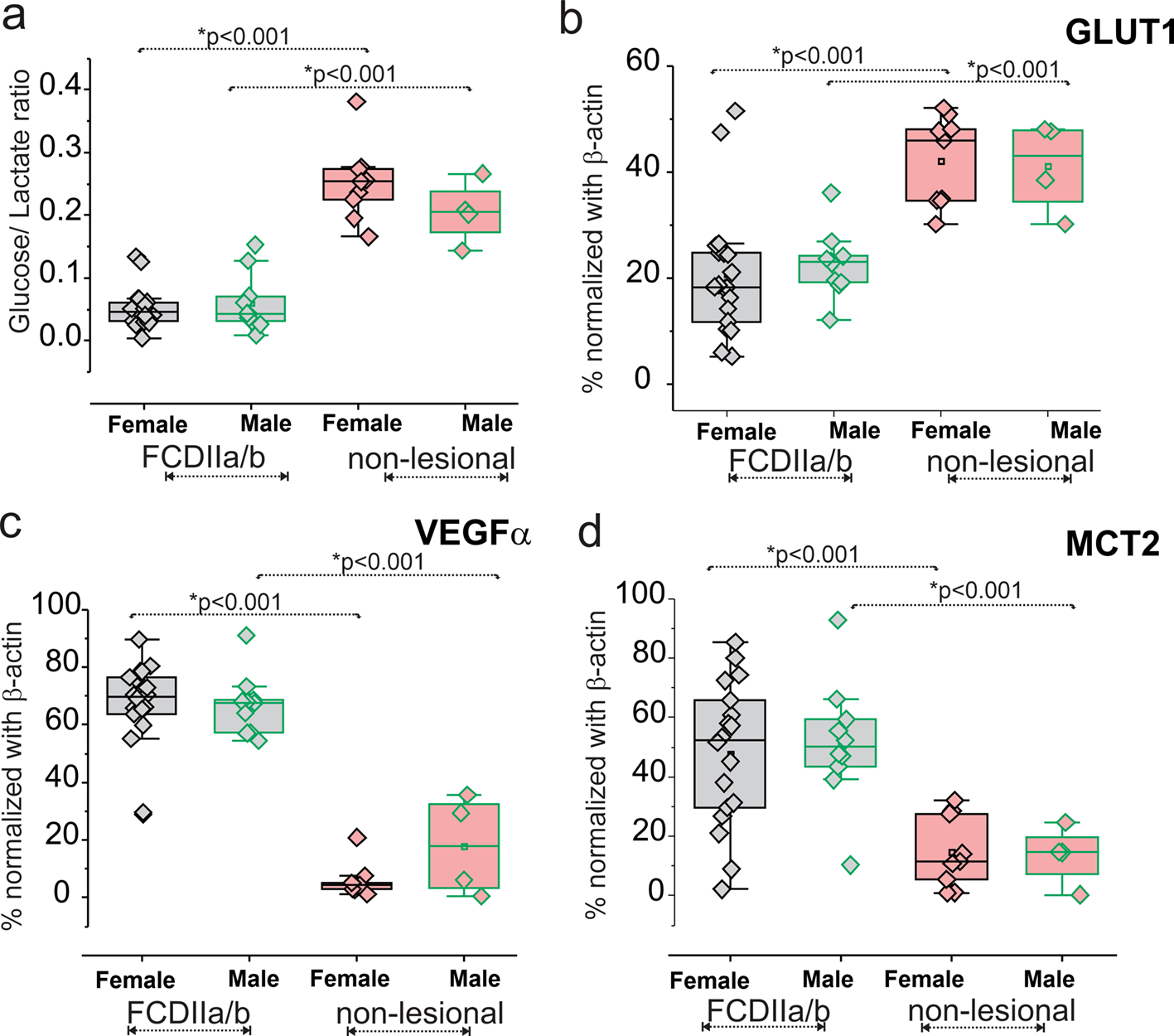

Supplement: Supplementary file 4 — GLUT1 suppression and low glucose-lactate ratios correspond to VEGFα elevation in FCDIIa and FCDIIb brain tissues is independent of gender. In both male and females in FCDIIa/b vs. non-lesional (a-d) showed decreased brain glucose-lactate ratio by biochemical analysis (a), suppressed GLUT1 levels (b), and upregulated VEGFα (c) and upregulated MCT2 (d) by western blot was observed across FCDIIa/b (male,n=10, female, n=21) compared to non-lesional (male, n=8, female, n=14). The quantified data of western blot normalized with loading control; β-actin is depicted (b-d). Values are mean ± SEM, ***p<0.001, two sample t-test. (PNG 323 KB) [file 12035_2025_4871_Fig7_ESM.png]

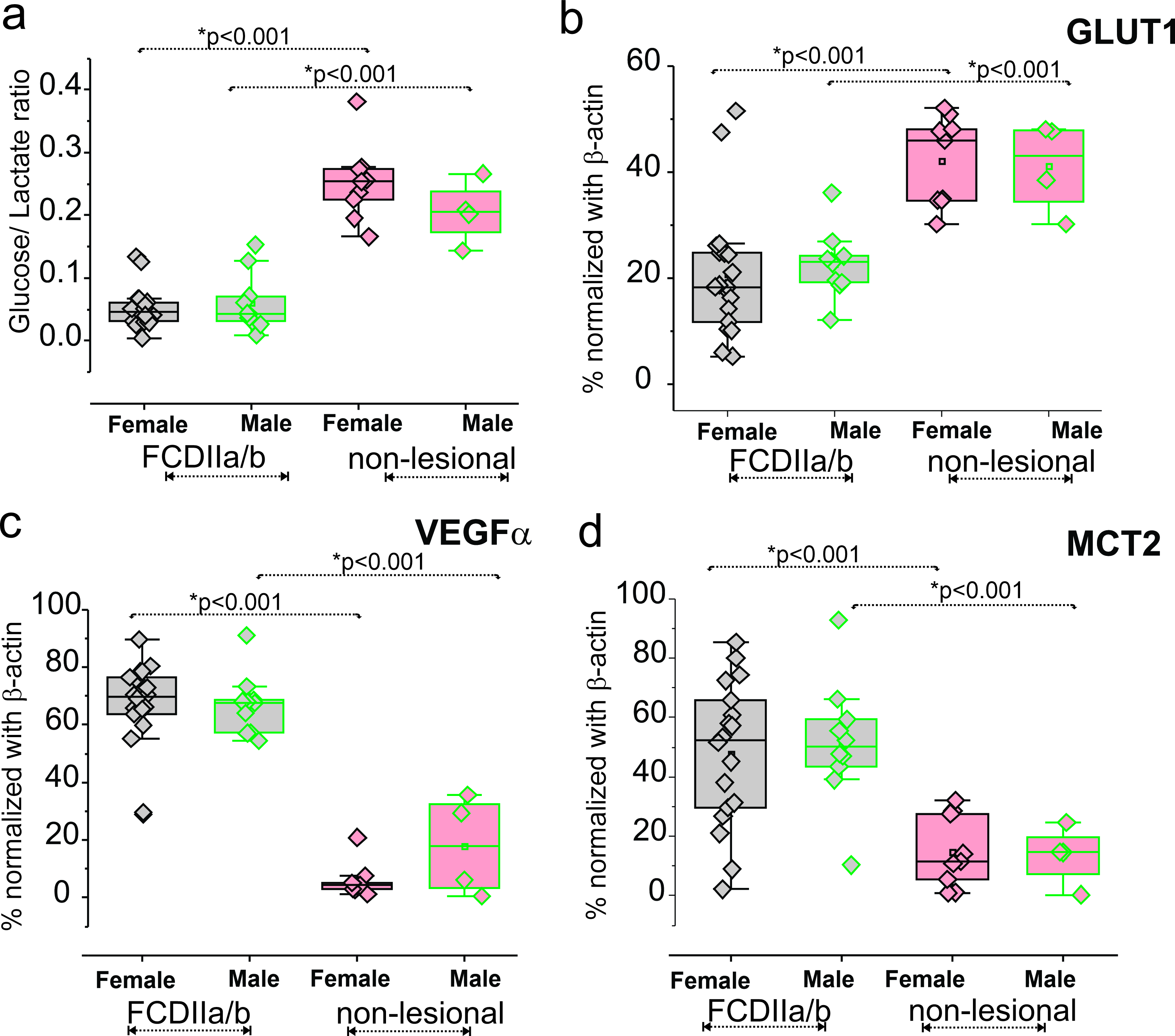

Supplement: Supplementary file 5 — High resolution image (TIF 33.2 MB) [file 12035_2025_4871_MOESM3_ESM.tif]

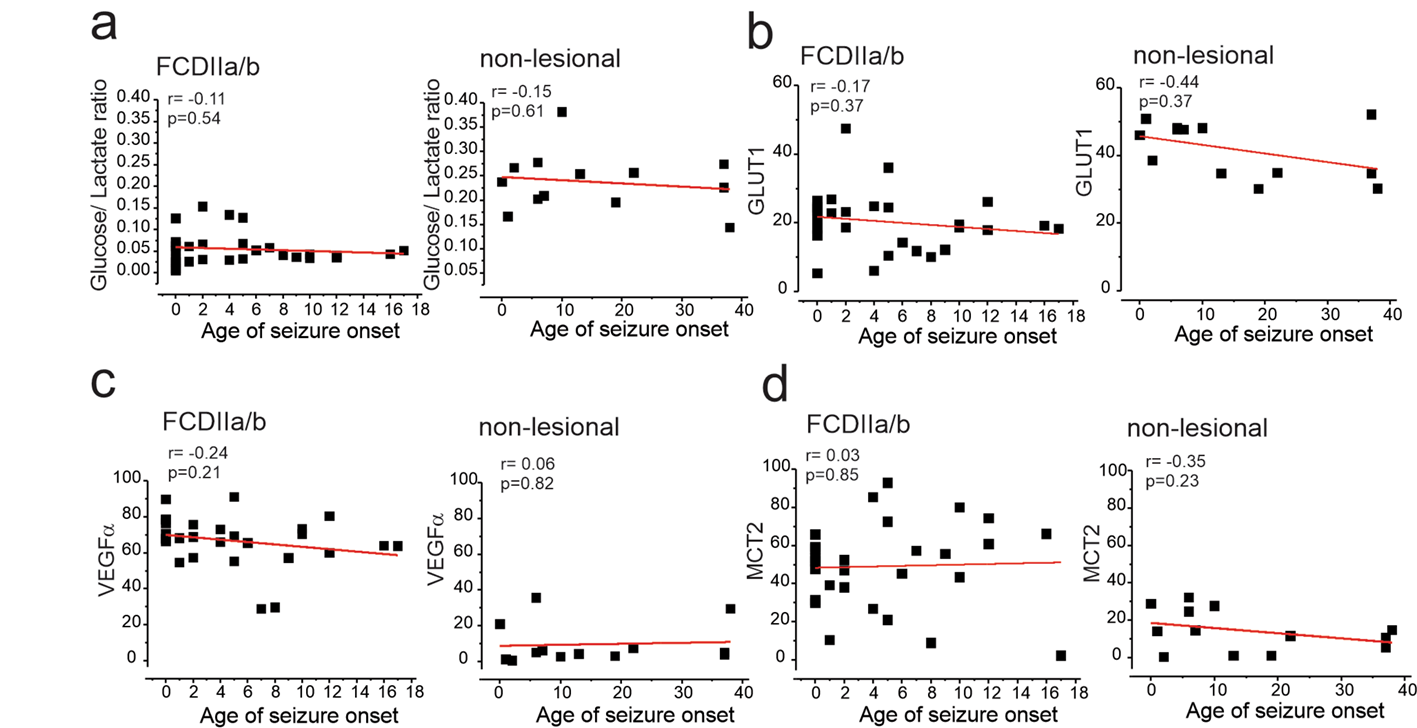

Supplement: Supplementary file 6 — Glucose/lactate, GLUT1, VEGFα, MCT2 levels in the FCDIIa/b is not correlated to age of seizure onset. (a-d) Non-significant correlations were obtained for (a) Glucose/lactate (p = 0.54; r = -0.11) in FCDIIa/b and (p = 0.61; r = -0.15) in non-lesional; (b) GLUT1 (p = 0.37; r = -0.17) in FCDIIa/b and (p = 0.37;r = -0.44) in non-lesional; (c) VEGFα (p = 0.21; r = -0.24) in FCDIIa/b and (p = 0.82; r = 0.06) in non-lesional and, (d) MCT2 (p = 0.85; r = 0.03) in FCDIIa/b and (p = 0.23; r = -0.35) in non-lesional. However, the trend shows an average low glucose/lactate ratio across FCDIIa/b individuals regardless of their age of seizure and higher glucose/lactate ratio across non-lesional. Similarly, low GLUT1, high VEGFα and MCT2 is prominent in FCDIIa/b and reverse in non-lesional cases in correlation to age of seizure onset. (PNG 211 KB) [file 12035_2025_4871_Fig8_ESM.png]

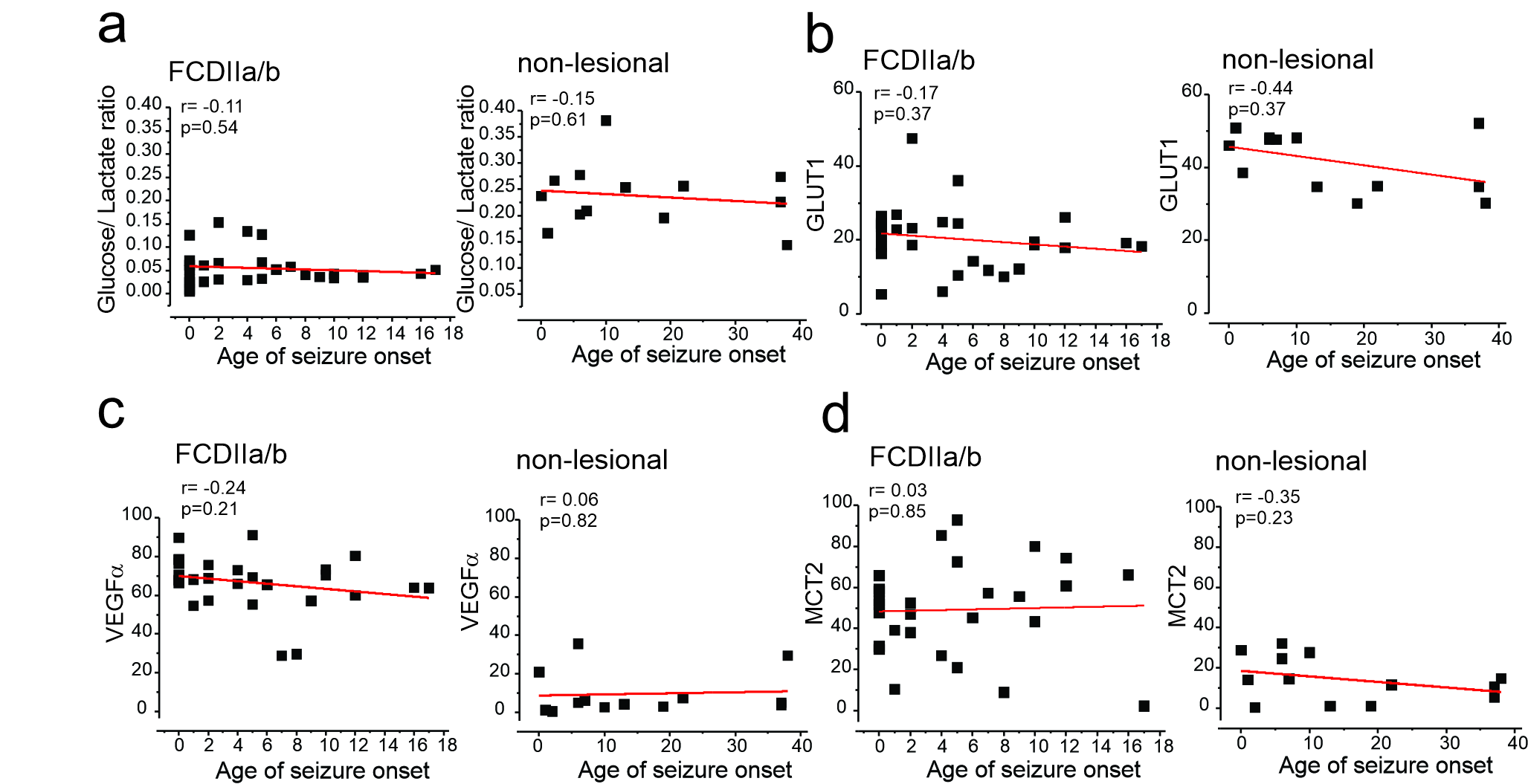

Supplement: Supplementary file 7 — High resolution image (TIF 8.80 MB) [file 12035_2025_4871_MOESM4_ESM.tif]

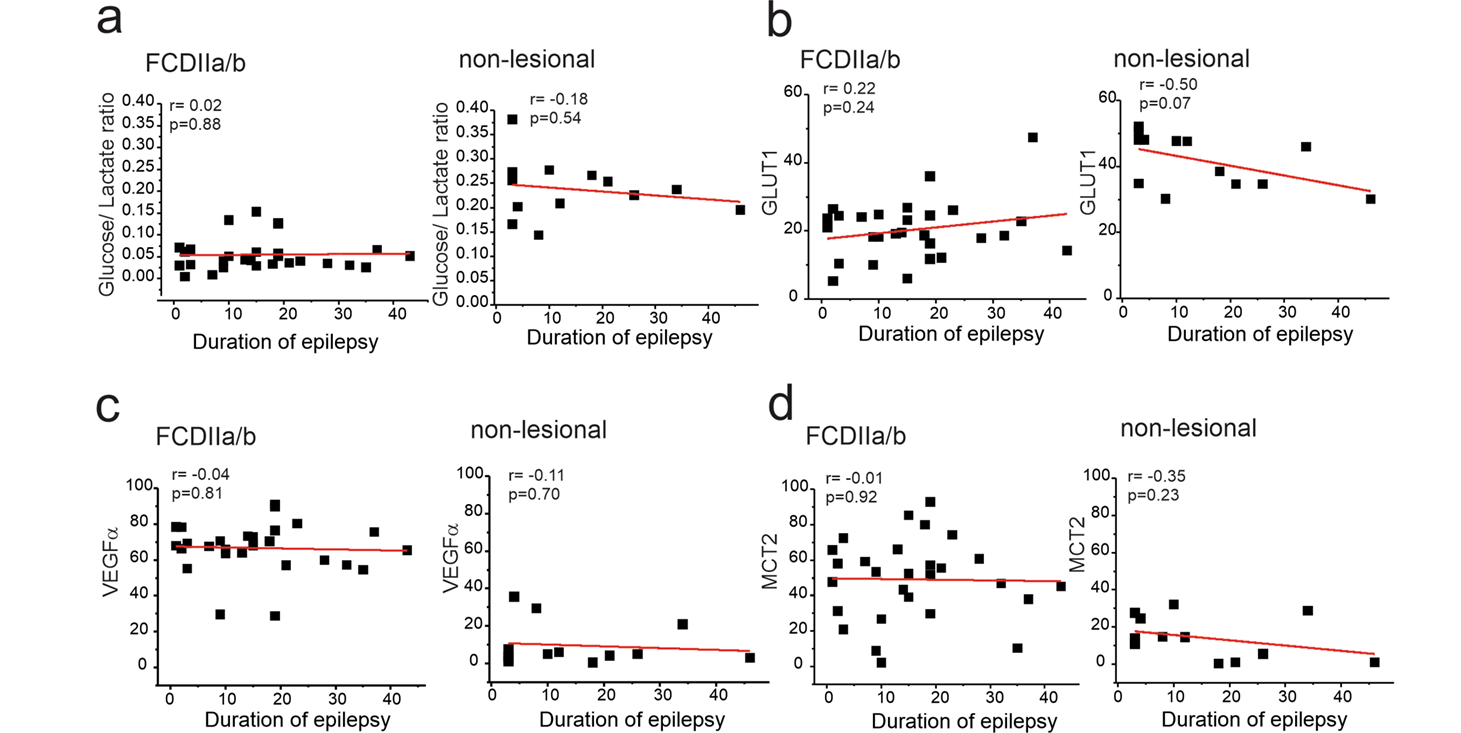

Supplement: Supplementary file 8 — Glucose/lactate, GLUT1, VEGFα, MCT2 levels in the FCDIIa/b is not correlated to duration of epilepsy. (a-d) Non-significant correlations were obtained for (a) Glucose/lactate (p= 0.88; r = 0.22) in FCDIIa/b and (p = 0.54; r = -0.18) in non-lesional; (b) GLUT1 (p = 0.24; r = 0.22) in FCDIIa/b and (p = 0.07; r = -0.50) in non-lesional; (c) VEGFα (p = 0.81; r = -0.04) in FCDIIa/b and (p = 0.70; r = 0.11) in non-lesional and, (d) MCT2 (p = 0.92; r = -0.01) in FCDIIa/b and (p = 0.23; r = -0.35) in non-lesional. However, the trend shows an average low glucose/lactate ratio across FCDIIa/b individuals regardless of their duration of epilepsy and higher glucose/lactate ratio across non-lesional individuals. Similarly, low GLUT1, high VEGFα and MCT2 is prominent in FCDIIa/b and reverse trend noted in non-lesional cases in correlation to duration of epilepsy. (PNG 194 KB) [file 12035_2025_4871_Fig9_ESM.png]

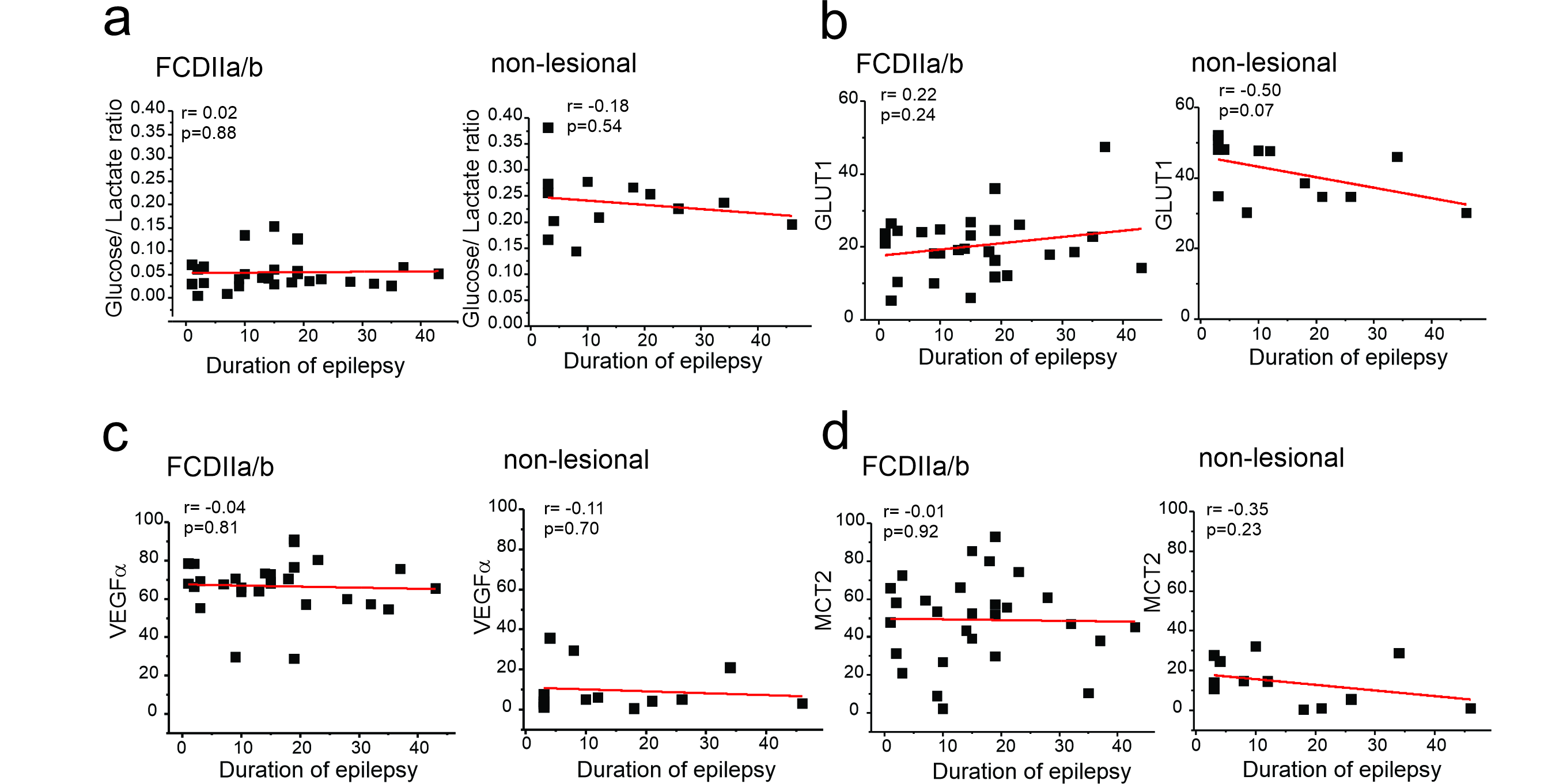

Supplement: Supplementary file 9 — High resolution image (TIF 14.6 MB) [file 12035_2025_4871_MOESM5_ESM.tif]

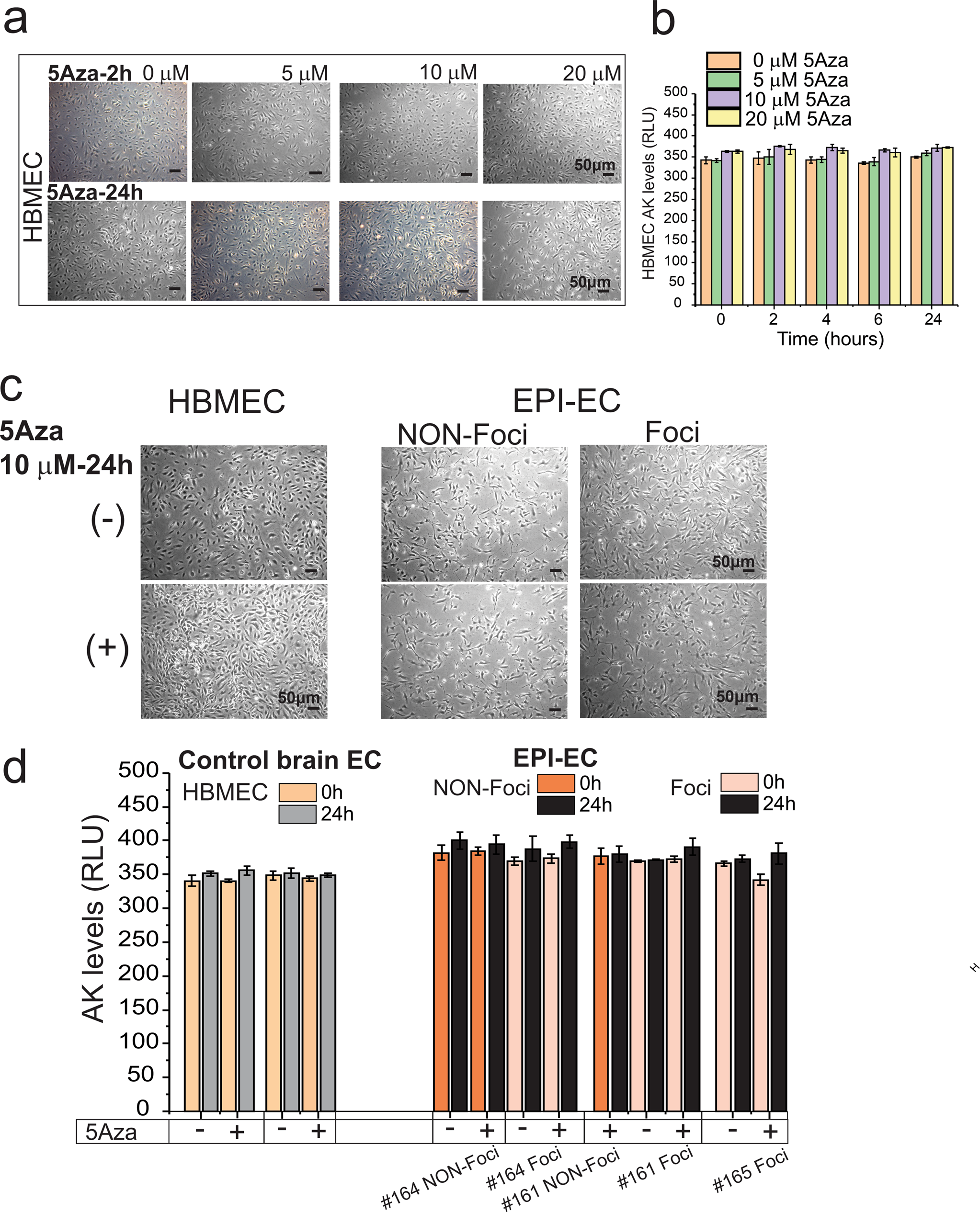

Supplement: Supplementary file 10 — Negligible cytotoxicity of DNA methylation inhibitor, 5Aza (decitabine) in human brain microvascular endothelial cells. (a-d) Cytotoxicity from 0, 2, 4, 6 and 24-h exposure to 5Aza at different concentrations (0, 5, 10 and 20 µM) on HBMEC/control ECs showed no significant difference in the levels of adenylate kinase (AK) released from damaged cells, measured in relative luminescence units (RLU). A representative phase-image of the HBMEC treated under different 5Aza concentration are also shown. Adenylate kinase (AK) levels within HBMECs and EPI-EC obtained from both foci/epileptic and non-foci/non-epileptic tissues showed non-significant difference with 24 h- 5Aza treatment. Phase-contrast microscope images (c) and AK levels by biochemical measurements (d) are shown, with and without 5Aza treatment among different brain EC types evaluated. Values are mean ± SEM, ***p<0.001, two-way ANOVA used for comparison. (PNG 2.48 MB) [file 12035_2025_4871_Fig10_ESM.png]

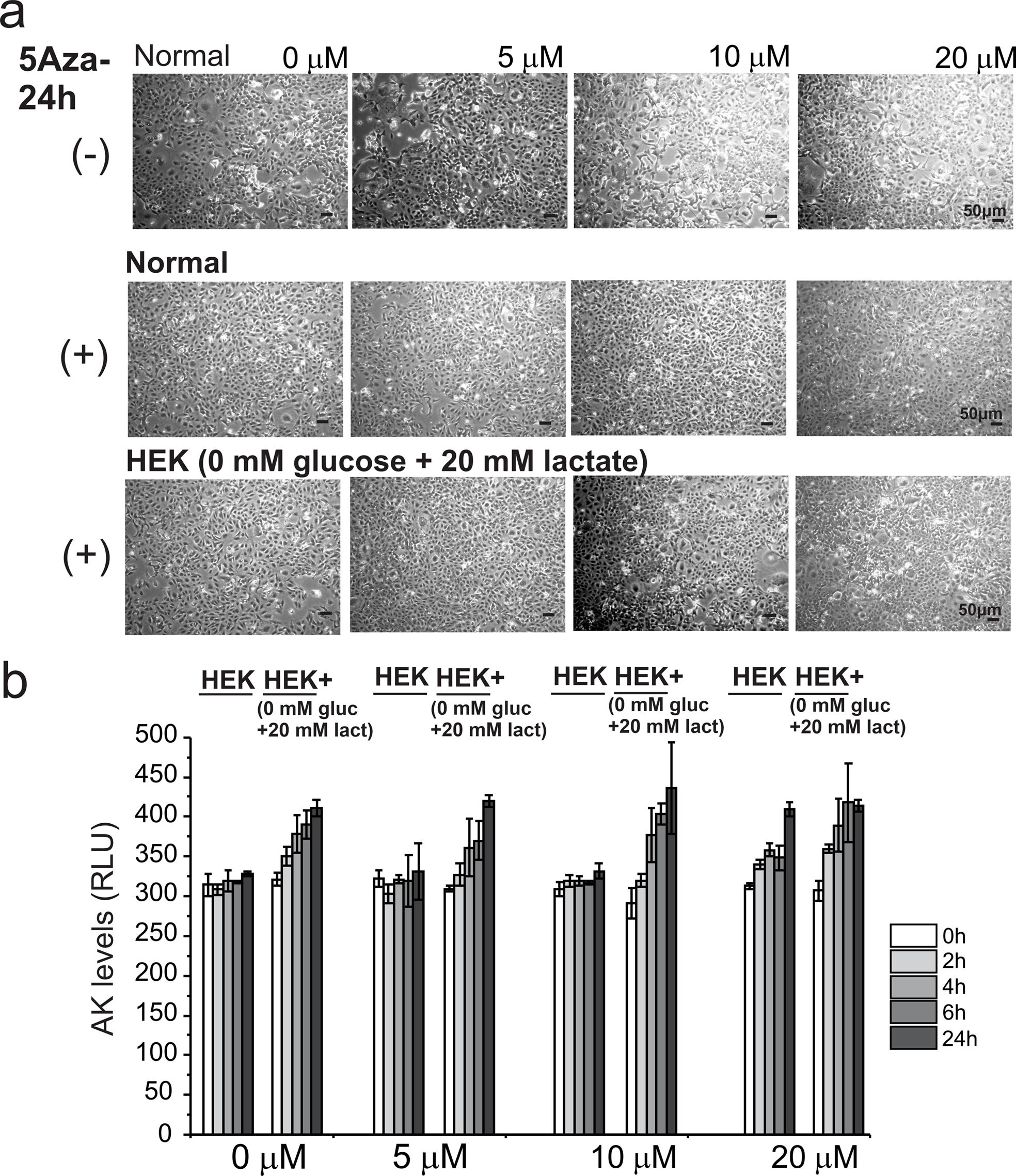

Supplement: Supplementary file 12 — DNA methylation inhibitor, decitabine (5Aza), pre- and post-conditioning with low glucose-high lactate showed no cytotoxicity in HEK cells. (a) Phase-contrast microscopy images showed minimal cell death in normal glucose and low glucose-high lactate conditions and after following concentration, 0, 5, 10, 20 µM 5Aza co-treatment. (b) AK levels is further suggestive of minimal cellular stress or non-damaged cells with 5Aza at different concentrations at 0 to 24 h, post-treatment. AK were measured in relative luminescence units (RLU) and compared with time and 5Aza-treatment concentrations. Values are mean ± SEM,***p<0.001, two-way ANOVA used for comparison (PNG 2.85 MB) [file 12035_2025_4871_Fig11_ESM.png]

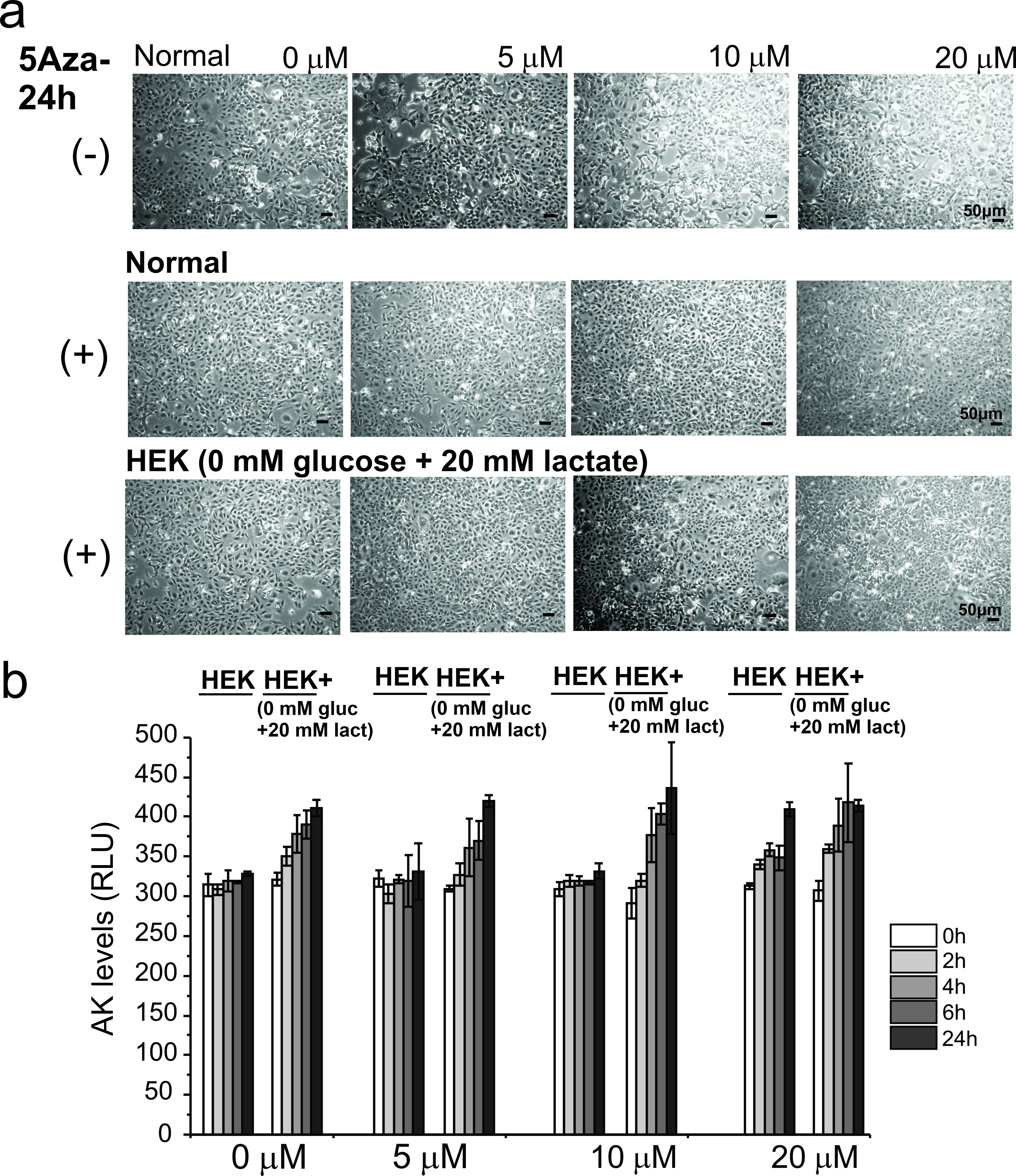

Supplement: Supplementary file 13 — High resolution image (TIF 59.5 MB) [file 12035_2025_4871_MOESM7_ESM.tif]

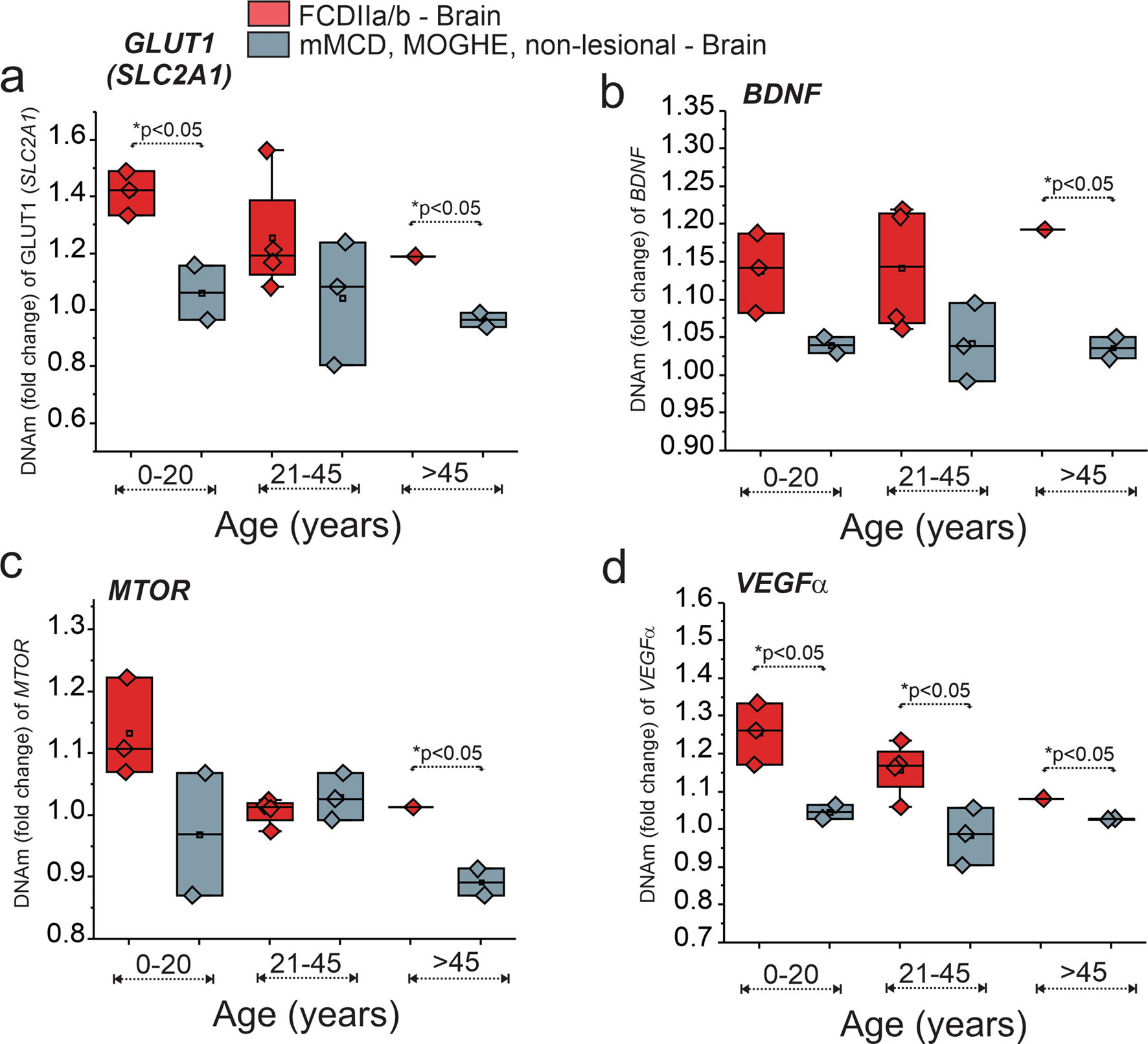

Supplement: Supplementary file 14 — Hypermethylation of GLUT1 (SLC2A1), and other key genes (BDNF, MTOR and VEGFα) in FCDIIa/b vs. other-FCD subtypes, mMCD, MOGHE and non-lesional brain tissues is independent of age. (a-d) In the small subject cohort, the DNA methylation of (a) GLUT1 (SLC2A1), (b) brain-derived neurotrophic factor (BDNF), (c) MTOR, and (d) VEGFα in brain of FCDIIa/b, mMCD, MOGHE and non-lesional tissues were compared to age of these individuals across age groups (0-20; 21-45 and above 45 years old). Fold-change using nominal p-value from generalized linear model shows gain of methylation in individuals with FCD subtypes for SLC2A1,BDNF, and MTOR, VEGFα using t-test (*p < 0.05) for comparison (PNG 418 KB) [file 12035_2025_4871_Fig12_ESM.png]

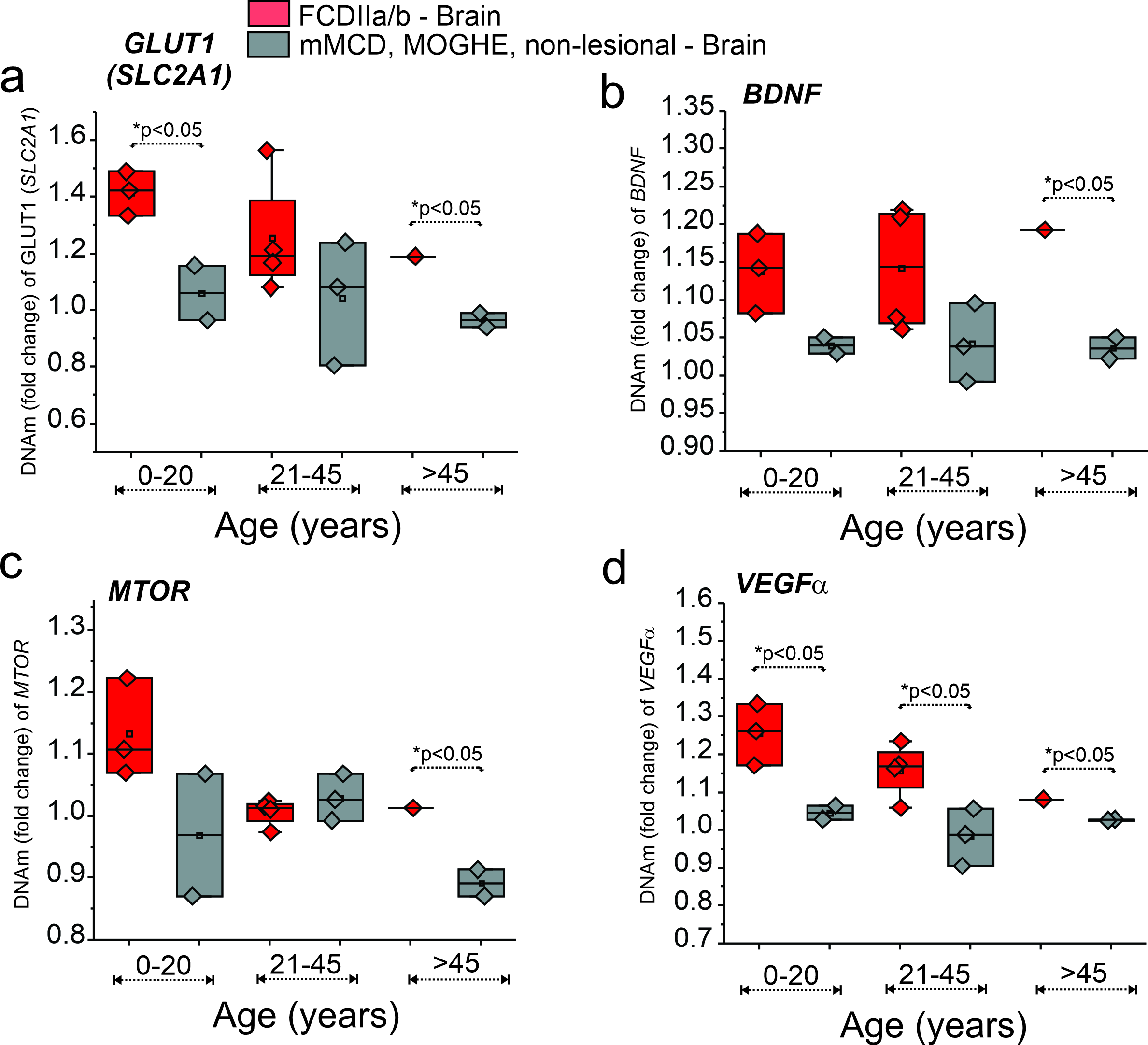

Supplement: Supplementary file 15 — High resolution image (TIF 20.1 MB) [file 12035_2025_4871_MOESM8_ESM.tif]

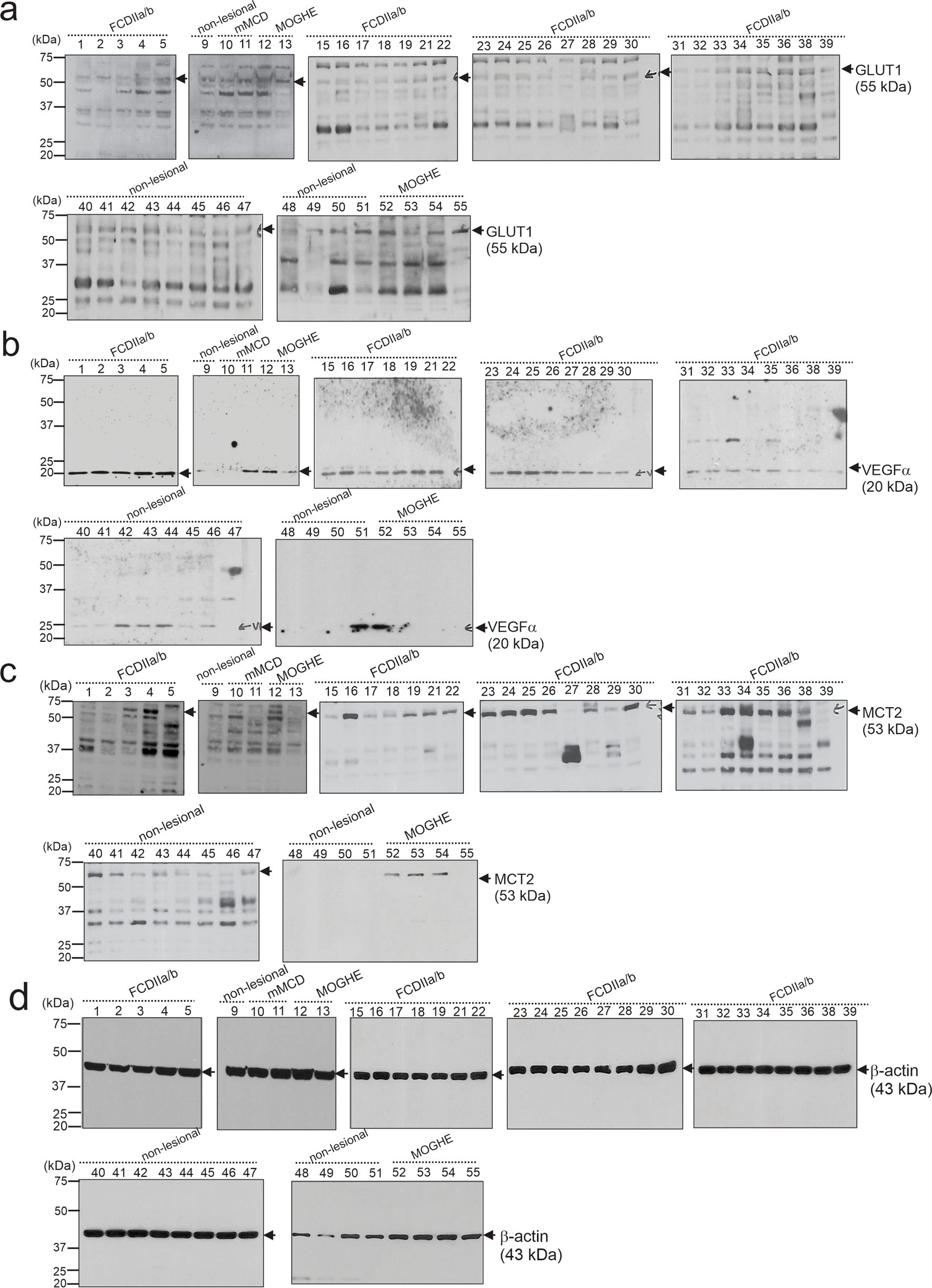

Supplement: Supplementary file 16 — Full Representative western blots of GLUT1, VEGFα, MCT2 and β-actin in FCD subtypes, FCDIIa/b, mMCD, MOGHE and non-lesional brain tissue samples (e.g., #ID 1-55) blots are shown (PNG 1.88 MB) [file 12035_2025_4871_Fig13_ESM.png]

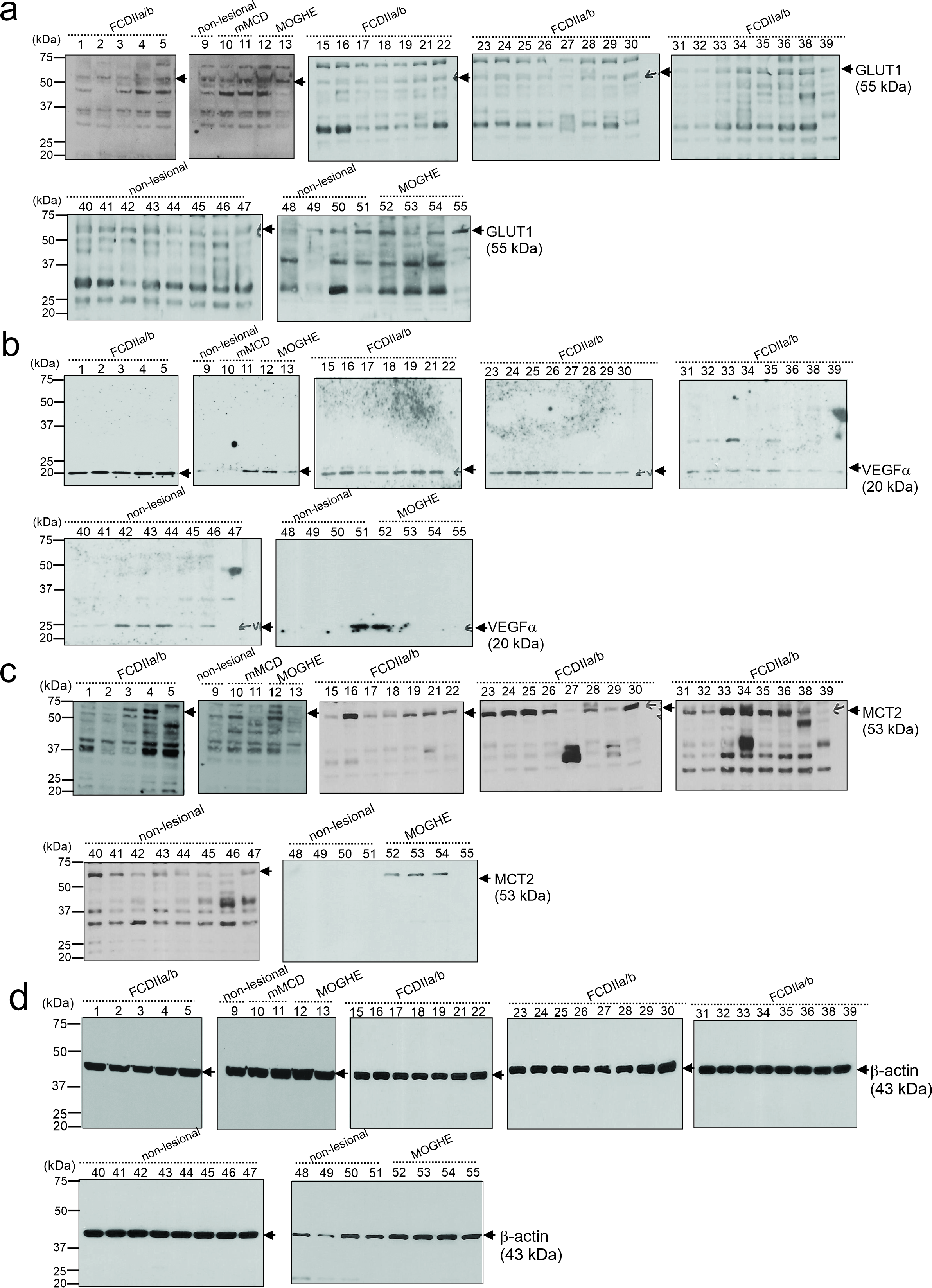

Supplement: Supplementary file 17 — High resolution image (TIF 30.4 MB) [file 12035_2025_4871_MOESM9_ESM.tif]

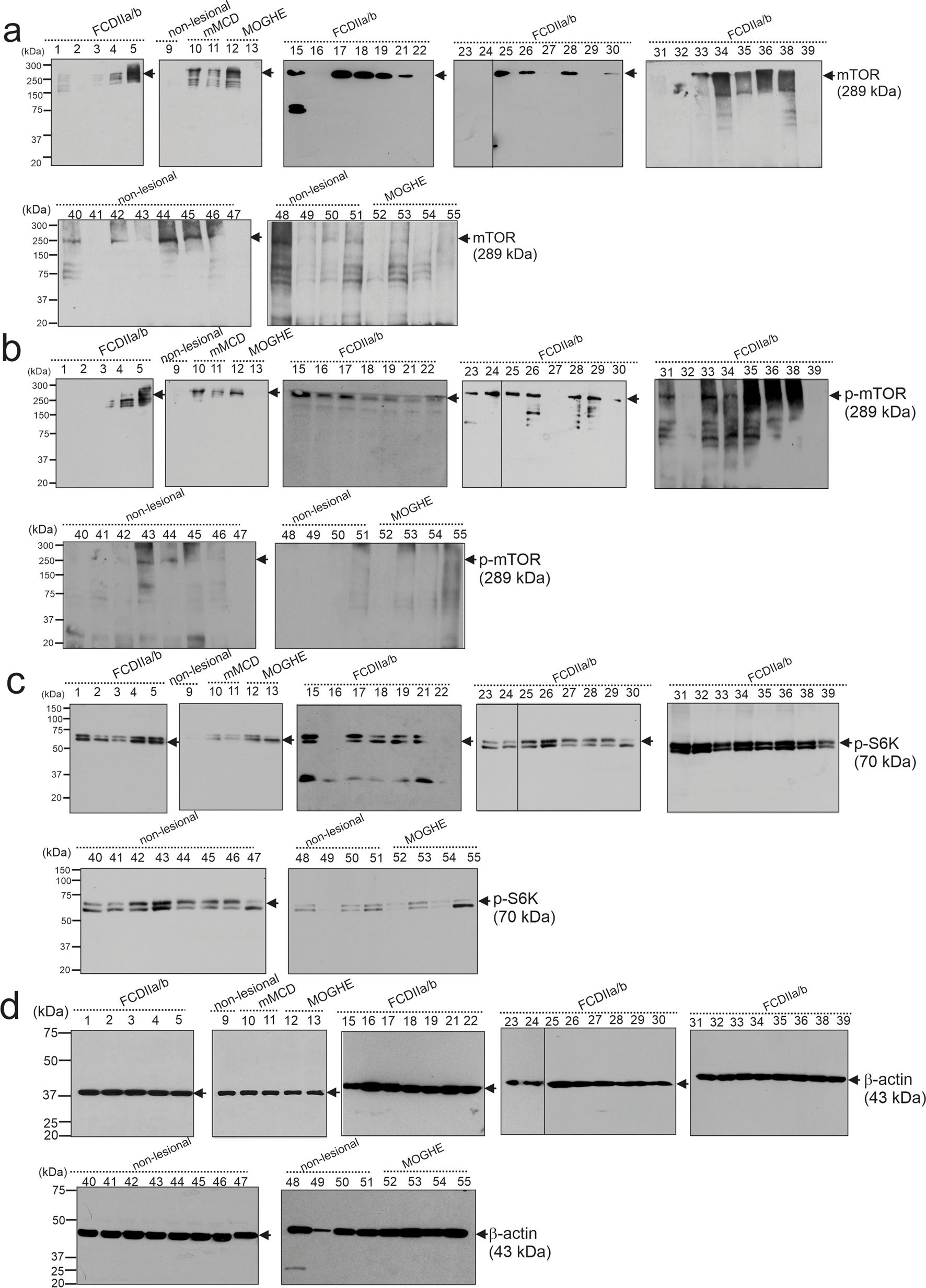

Supplement: Supplementary file 18 — Full Representative western blots of mTOR, p-mTOR, p-SK6 and respective β-actin in FCD subtypes, FCDIIa/b, mMCD, MOGHE and non-lesional brain tissue samples (e.g., #ID 1-55) blots are shown (PNG 2.18 MB) [file 12035_2025_4871_Fig14_ESM.png]

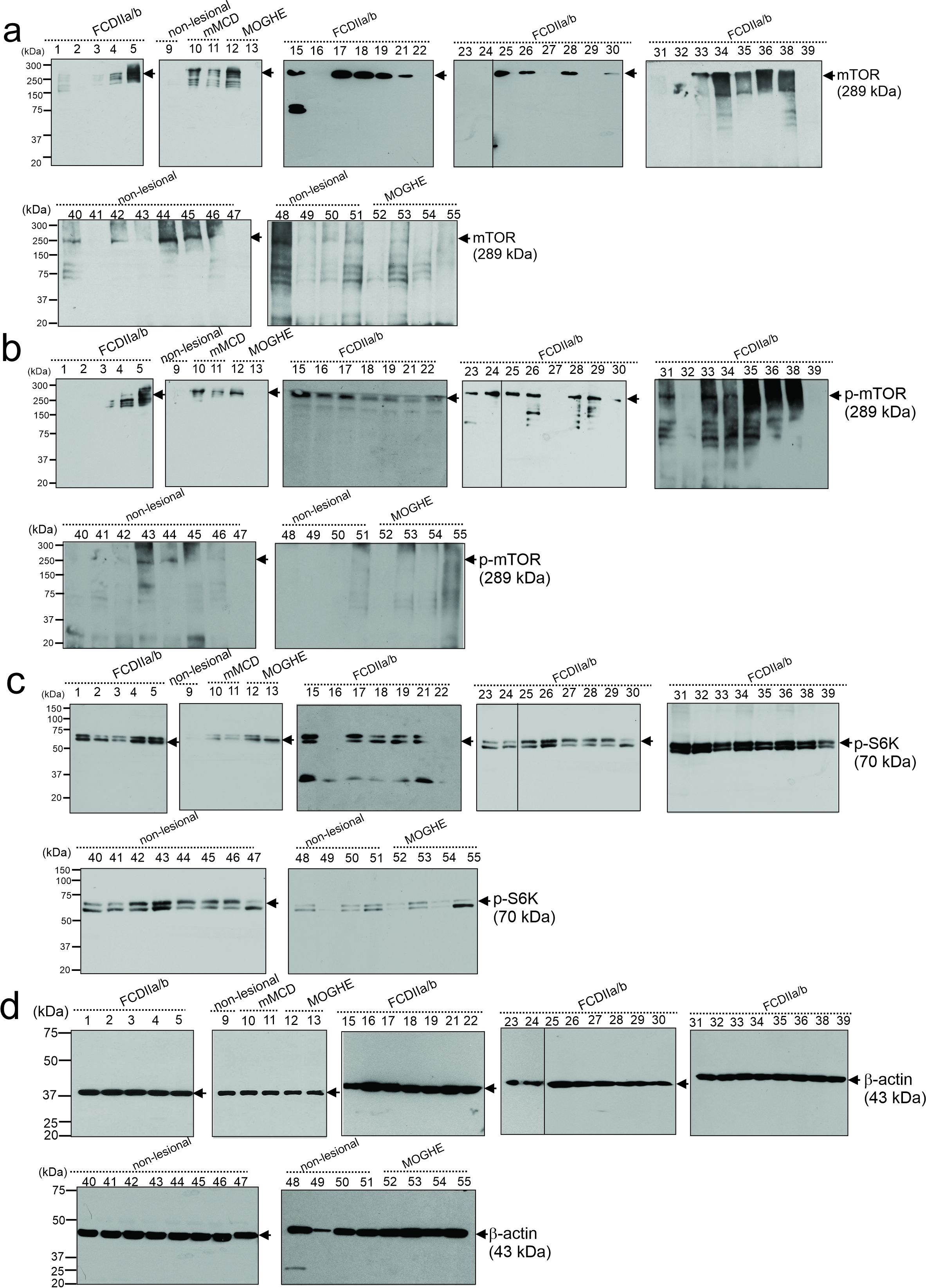

Supplement: Supplementary file 19 — High resolution image (TIF 30.2 MB) [file 12035_2025_4871_MOESM10_ESM.tif]

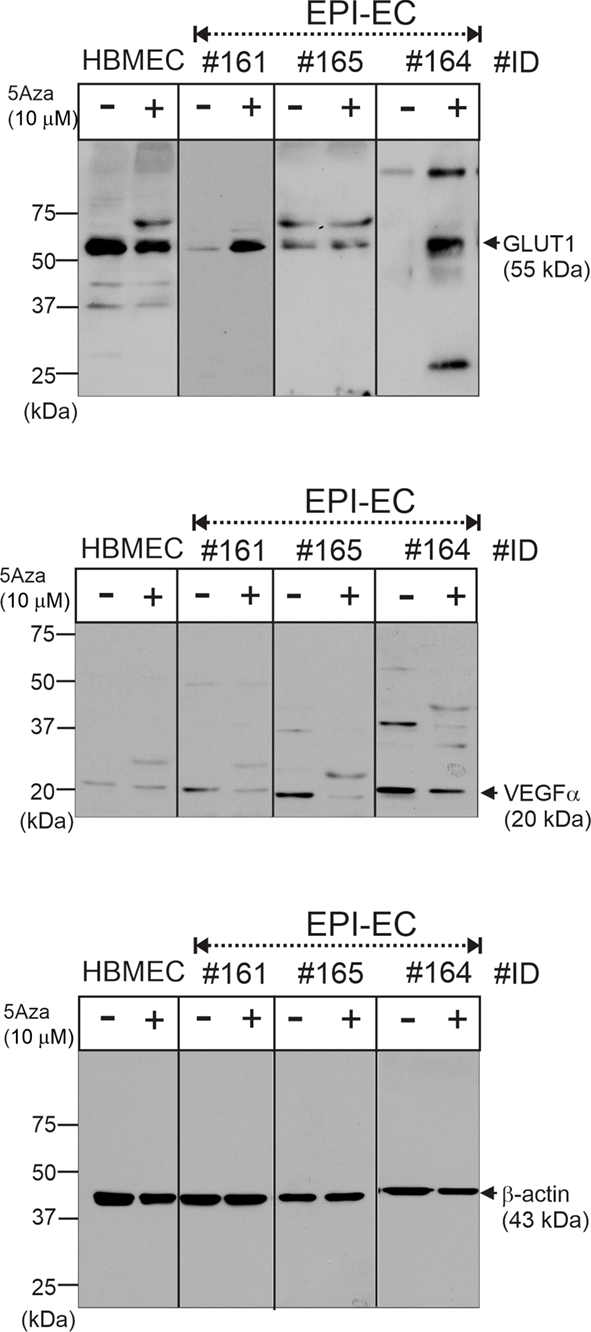

Supplement: Supplementary file 20 — Full Representative western blots: GLUT1, VEGFα and β-actin post DNA methylation inhibitor 5Aza (decitabine) treatment in EPI-ECs compared to untreated cells counterpart are shown. Individual EPI-EC from respective patients were denoted by ID #161, #165, #164 and compared with normal EC (HBMEC) (PNG 361 KB) [file 12035_2025_4871_Fig15_ESM.png]

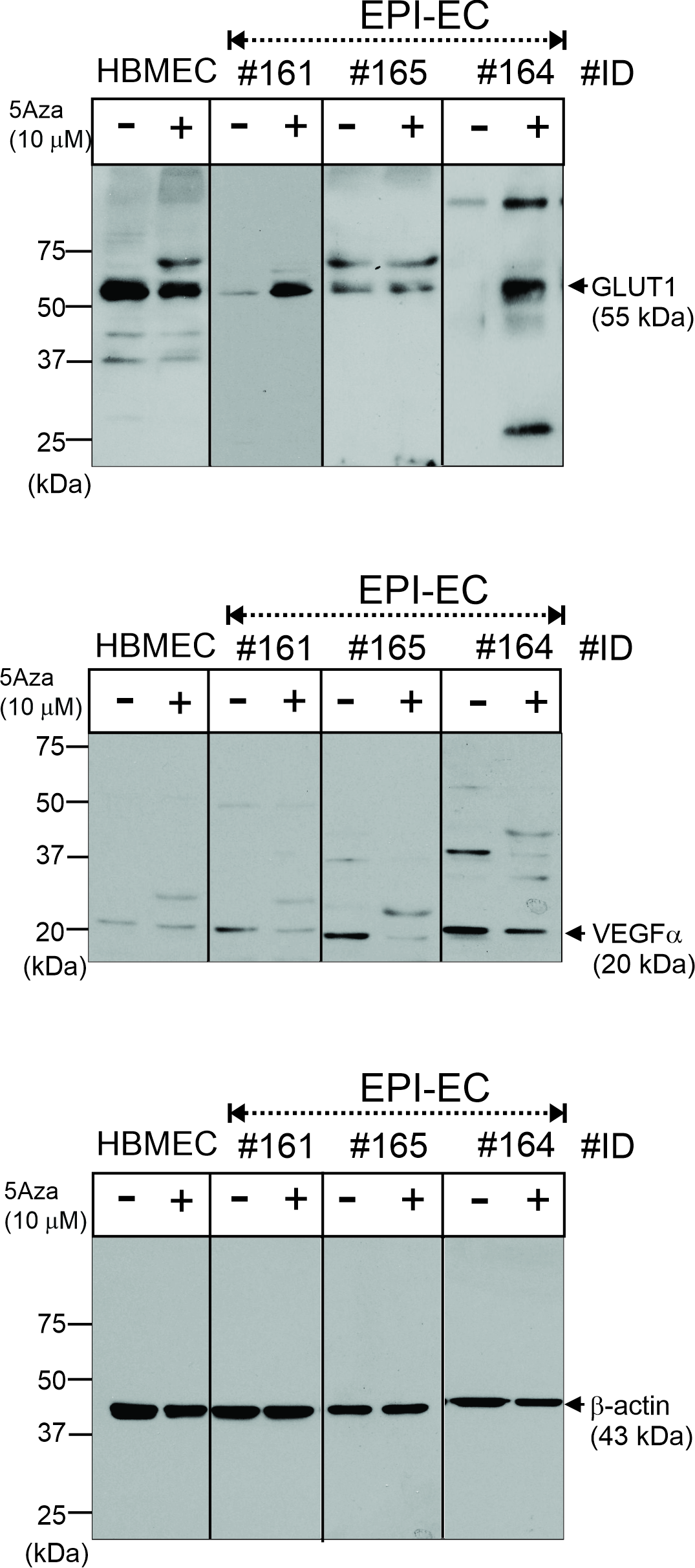

Supplement: Supplementary file 21 — High resolution image (TIF 10.4 MB) [file 12035_2025_4871_MOESM11_ESM.tif]

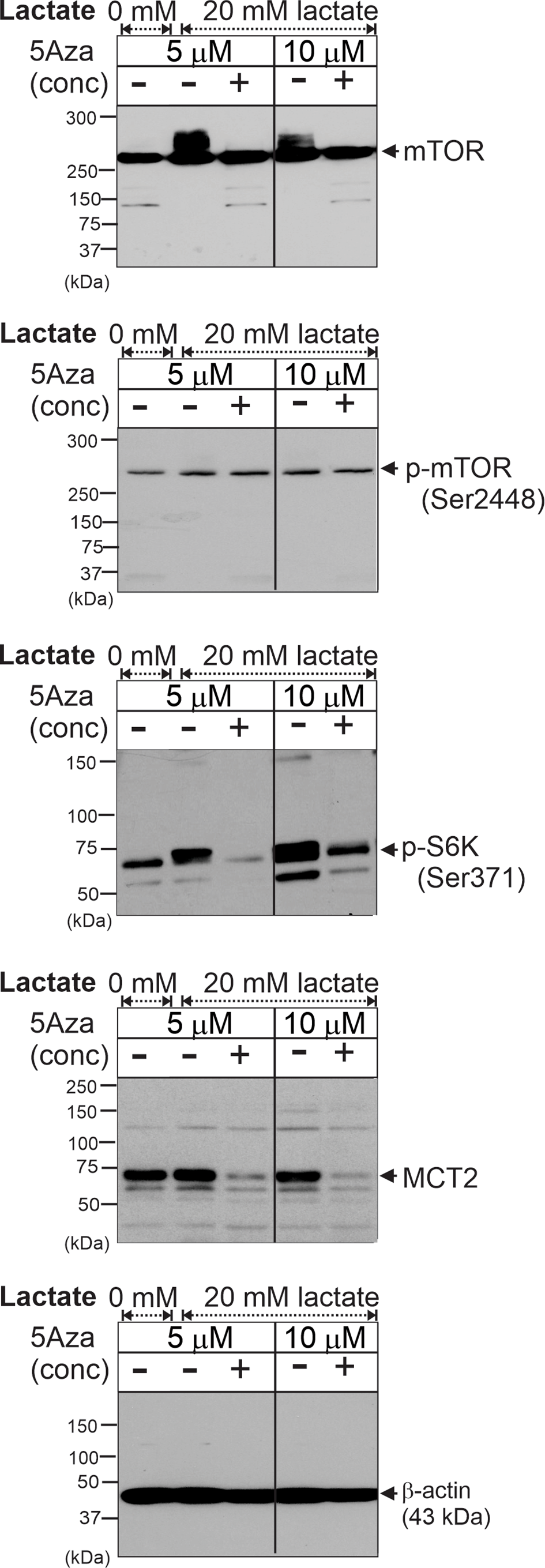

Supplement: Supplementary file 22 — Full Representative western blots of mTOR, p-mTOR, p-SK6, MCT2 and β-actin in HEK cells treated with low glucose-high lactate, and subsequently with or without inhibition of DNA methylation (5Aza) are shown (PNG 918 KB) [file 12035_2025_4871_Fig16_ESM.png]

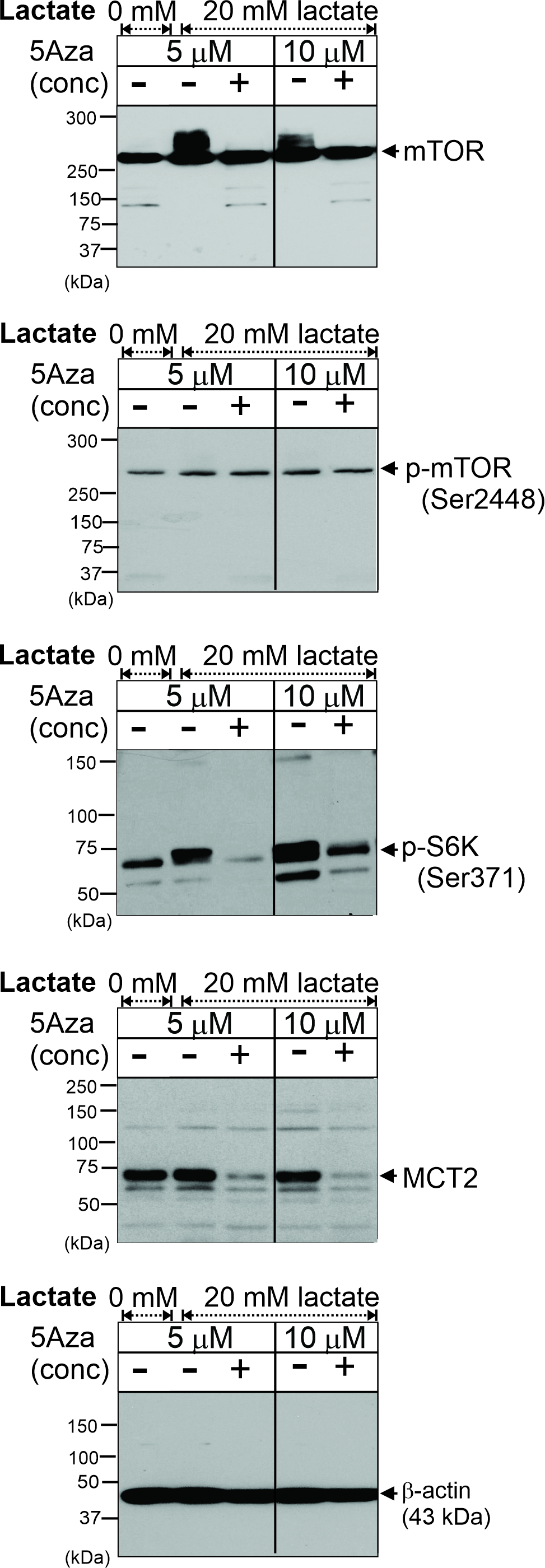

Supplement: Supplementary file 23 — High resolution image (TIF 10.9 MB) [file 12035_2025_4871_MOESM12_ESM.tif]
