# Supplementary material for: GLUT1 and Cerebral Glucose Hypometabolism in Human Focal Cortical Dysplasia Is Associated with Hypermethylation of Key Glucose Regulatory Genes
Source: Mol Neurobiol. 2025 Apr 7;62(8):10264–76. doi: 10.1007/s12035-025-04871-z (PMC12289428; doi:10.1007/s12035-025-04871-z)
Supplement: Supplementary file 24 — Supplementary file13 (DOCX 30 KB) [file 12035_2025_4871_MOESM13_ESM.docx]

| **#ID** | **Age**  (yrs) | **Sex** | **Age of seizure onset**  (yrs) | **Duration of epilepsy** (yrs) | **Years surgery to follow-up** | **Outcome**  **(Engel score)** | **Pathology** | **Invasive Evaluation** | **Experimental use** |
| --- | --- | --- | --- | --- | --- | --- | --- | --- | --- |
| 2a | 50 | M | 8 | 42 | 7.20 | Ia | FCDIIa | SEEG | DNAm |
| 3a | 25 | F | 13 | 12 | 5.75 | II | FCDIIb | Grids/Depths | DNAm |
| 4a | 28 | F | 13 | 15 | 1.30 | Ia | FCDIIb | Grids/Depths | DNAm |
| 6a | 53 | M | 49 | 4 | 5.71 | III | non-lesional | SEEG | DNAm |
| 7a | 49 | M | 36 | 13 | 5.56 | Ic | non-lesional | No | DNAm |
| 1 | 14 | F | 4 | 10 | 6.75 | Id | FCD IIa | SEEG | DNAm, WB |
| 2 | 19 | F | 0 | 19 | 2.25 | unknown | FCD IIa | SEEG | DNAm, WB |
| 3 | 17 | M | 2 | 15 | 2.96 | II | FCD IIa | No | DNAm, WB |
| 4 | 24 | M | 5 | 19 | 1.01 | II | FCD IIb | No | DNAm, WB |
| 5 | 39 | F | 2 | 37 | 0.26 | Ia | FCD IIb | SEEG | DNAm, WB |
| 9 | 34 | F | 0 | 34 | 4.77 | III | non-lesional | SEEG | DNAm, WB |
| 10 | 16 | M | 5 | 11 | 4.98 | II | mMCD | SEEG | DNAm, WB |
| 11 | 34 | F | 23 | 11 | 4.70 | Ib | mMCD | No | DNAm, WB |
| 12 | 28 | F | 20 | 8 | 5.43 | Ib | MOGHE | SEEG | DNAm, WB |
| 13 | 8 | M | 0 | 8 | 0.19 | Ia | MOGHE | SEEG | DNAm, WB |
| 15 | 24 | M | 10 | 14 | 0.06 | unknown | FCD IIa | SEEG | WB |
| 16 | 35 | F | 12 | 23 | 4.57 | I | FCD IIa | Grids/Depths | WB |
| 17 | 8 | F | 5 | 3 | 0.98 | Ia | FCD IIa | SEEG | WB |
| 18 | 34 | M | 2 | 32 | 9.50 | II | FCD IIa | No | WB |
| 19 | 40 | F | 12 | 28 | 0.35 | unknown | FCD IIa | SEEG | WB |
| 20 | 22 | F | 14 | 8 | 6.55 | II | FCD IIa | Grids/Depths | WB |
| 21 | 7 | M | 0 | 7 | 0.54 | IV | FCD IIa | No | WB |
| 22 | 2 | F | 0 | 2 | 7.15 | Ia | FCD IIa | No | WB |
| 23 | 9 | F | 0 | 9 | 3.78 | Ia | FCD IIa | Grids/Depths | WB |
| 24 | 1 | F | 0 | 1 | 3.99 | Ia | FCD IIa | No | WB |
| 25 | 28 | F | 10 | 18 | 2.28 | III-IV | FCD IIa | Grids/Depths | WB |
| 26 | 27 | F | 17 | 10 | 7.55 | II | FCD IIa | SEEG | WB |
| 27 | 19 | F | 0 | 19 | 2.25 | unknown | FCD IIa | SEEG | WB |
| 28 | 36 | M | 1 | 35 | 0.77 | Ia | FCD IIb | SEEG | WB |
| 29 | 1 | M | 0 | 1 | 3.36 | Ia | FCD IIb | No | WB |
| 30 | 16 | M | 1 | 15 | 2.51 | III | FCD IIb | No | WB |
| 31 | 2 | F | 0 | 2 | 1.60 | II | FCD IIb | No | WB |
| 32 | 19 | F | 4 | 15 | 3.01 | Ia | FCD IIb | No | WB |
| 33 | 8 | F | 5 | 3 | 7.14 | Ia | FCD IIb | No | WB |
| 34 | 49 | F | 6 | 43 | 9.85 | Ia | FCD IIb | No | WB |
| 35 | 30 | M | 9 | 21 | 5.76 | Ib | FCD IIb | SEEG | WB |
| 36 | 29 | M | 16 | 13 | 8.04 | Ia | FCD IIb | Grids/Depths | WB |
| 37 | 6 | F | 0 | 6 | 0.34 | III | FCD IIb | No | WB |
| 38 | 26 | F | 7 | 19 | 4.38 | Ia | FCD IIb | No | WB |
| 39 | 17 | F | 8 | 9 | 7.80 | Ib | FCD IIb | SEEG | WB |
| 40 | 16 | F | 6 | 10 | 2.53 | II | Non-lesional | SEEG | WB |
| 41 | 13 | F | 10 | 3 | 8.18 | unknown | Non-lesional | SEEG | WB |
| 42 | 19 | M | 7 | 12 | 2.39 | II | Non-lesional | Grids/Depths | WB |
| 43 | 10 | M | 6 | 4 | 6.40 | IV | Non-lesional | Grids/Depths | WB |
| 44 | 46 | M | 38 | 8 | 3.23 | I | Non-lesional | Grids/Depths | WB |
| 45 | 4 | F | 1 | 3 | 2.20 | III- IV | Non-lesional | No | WB |
| 46 | 25 | F | 22 | 3 | 2.45 | IV | Non-lesional | No | WB |
| 47 | 40 | F | 37 | 3 | 8.15 | Ia | Non-lesional | No | WB |
| 48 | 34 | F | 13 | 21 | 7.73 | Ia | Non-lesional | SEEG | WB |
| 49 | 65 | F | 19 | 46 | 7.88 | Ia | Non-lesional | SEEG | WB |
| 50 | 20 | M | 2 | 18 | 5.67 | Id | Non-lesional | SEEG | WB |
| 51 | 63 | F | 37 | 26 | 2.16 | III | Non-lesional | SEEG | WB |
| 52 | 51 | F | 3 | 48 | 0.57 | III | MOGHE | No | WB |
| 53 | 2 | M | 0 | 2 | 1.30 | III | MOGHE | No | WB |
| 54 | 17 | F | 13 | 4 | 1.59 | Ia | MOGHE | No | WB |
| 55 | 8 | M | 0 | 8 | 0.19 | Ia | MOGHE | SEEG | WB |
| P161 | 16 | M | 3 | 13 | 1.30 | Ia | FCDIIb | No | Cell culture experiments |
| P165 | 44 | F | 11 | 33 | 2.56 | Ic | FCD | No | Cell culture experiments |
| P164 | 67 | M | 30 | 37 | 2.49 | Ia | FCD | SEEG | Cell culture experiments |
|  | | | | | | | | | |

**Supplemental Table 1: Demographic and Clinical details.**

Abbreviations: Years, Yrs; Female, F; Male, M; Seizure Outcome/Engel score: I (seizure-free), II (rare seizures), III (worthwhile improvement), IV (no worthwhile improvement); focal cortical dysplasia, FCD; Stereo-EEG, SEEG; Western blot, WB; DNA methylation, DNAm.
