# Supplementary material for: GLUT1 and Cerebral Glucose Hypometabolism in Human Focal Cortical Dysplasia Is Associated with Hypermethylation of Key Glucose Regulatory Genes
Source: Mol Neurobiol. 2025 Apr 7;62(8):10264–76. doi: 10.1007/s12035-025-04871-z (PMC12289428; doi:10.1007/s12035-025-04871-z)
Supplement: Supplementary file 25 — Supplementary file14 (DOCX 23 KB) [file 12035_2025_4871_MOESM14_ESM.docx]

**Supplemental Table 2.** List of antibodies used for western blot.

(**a**)

| **Primary Antibody** | **Host** | **Concentration** | **Manufacturer** | **Catalog No.** |
| --- | --- | --- | --- | --- |
| GLUT1 | Rabbit | 1:500 | Abcam, Cambridge, MA | ab652  ab115730 |
| VEGFα | Mouse | 1:100 | Santa Cruz Biotechnology,  Inc., TX | sc-7269 |
| MCT2 | Rabbit | 1:1000 | Invitrogen, Thermo Fisher Scientific, Carlsbad, CA | PA5-112712 |
| mTOR substrates antibody kit  includes, mTOR  p-mTOR (Ser2448)  and p-S6K(Ser371) | Rabbit | 1:1000 | Cell Signaling Technology,  Danvers, MA | 9862 |
| β-actin | Mouse | 1:10,000 | Sigma-Aldrich, St. Louis,  MO | A1978 |

(**b**)

| **Secondary Antibody** | **Concentration** | **Manufacturer** | **Catalog No.** |
| --- | --- | --- | --- |
| Polyclonal Goat Anti-Rabbit IgG HRP | 1:2500 | Dako, part of ThermoFisher Scientific Waltham, MA | P0448 |
| Polyclonal Goat Anti-Mouse IgG HRP | 1:2500 | Dako, part of ThermoFisher Scientific Waltham, MA | P0447 |
